# Supplementary material for: CAR T cell engineering impacts antigen-independent activation and co-inhibition
Source: Mol Ther Methods Clin Dev. 2025 Sep 3;33(4):101586. doi: 10.1016/j.omtm.2025.101586 (PMC12464704; doi:10.1016/j.omtm.2025.101586)
Supplement: Document S1. Figures S1–S5 and Table S1 [file mmc1.pdf]

OMTM, Volume *33*

Supplemental information

CAR T cell engineering impacts antigen-independent activation and co-inhibition

Christoph Schultheiß, Simon Stücheli, Brenda Besemer, Maja Kadel, Paul Schmidt-Barbo, Andreas Zingg, Natascha Franz, Sarah Adamo, Frank Stenner, Claudia Fischer, Heinz Läubli, and Mascha Binder

Table S1. Antibodies used for flow cytometry.

Target	Conjugated fluorophore	Supplier	Reference Number	Host species	Comment
EGFRt	NA	Merck (Darmstadt, Germany)	NA (Erbitux®)	NA (Humanized)	Cetuximab, used to detect the spliced off truncated EGFR as a surrogate for the CAR construct. Diluted at 1:500.
Human IgG	FITC	Thermo Fisher Scientific	F4512-1ML	Rabbit	Used to detect Cetuximab/anti-EGFR-antibody. Diluted at 1:1000.
Whitlow/218-scFv-Linker	NA	Cell Signaling Technologies (Danvers, MA, USA)	57710S	Rabbit	Used to detect anti-CD19 CAR.
G4S-scFv-Linker	NA	Cell Signaling Technologies	71645S	Rabbit	Used to detect anti-R110 CAR.
Rabbit IgG	AF488	Cell Signaling Technologies	4412S	Goat	Used to detect anti-scFv-Linker-antibodies.
CD19	PE/Dazzle	BioLegend (San Diego, CA, USA)	302251	Mouse	
CD19	APC	BioLegend	302212	Mouse	
CD3	AF700	BioLegend	300424	Mouse	
CD3	FITC	BD BioSciences (Franklin Lanes, NJ, USA)	345763	Mouse	Used for sorting prior to single-cell sequencing.
CD4	BV605	BioLegend	344646	Mouse	
CD8	BV650	BioLegend	344729	Mouse	
PD-1	PE	BioLegend	329905	Mouse	
LAG-3	PE/Cy7	BioLegend	369309	Mouse	
TIM-3	APC/Cy7	BioLegend	345025	Mouse	
CD69	APC	BioLegend	985206	Mouse	
CCR7	PerCP	Invitrogen (Carlsbad, CA, USA)	46-1979-42	Rat	
CD45RA	APC	Invitrogen	47-0458-42	Mouse	
IGLV3-21^{R110} light chain	PE	AVA LifeScience (Denzlingen, Germany)	AVA-D01-PE/01P0150	Mouse	

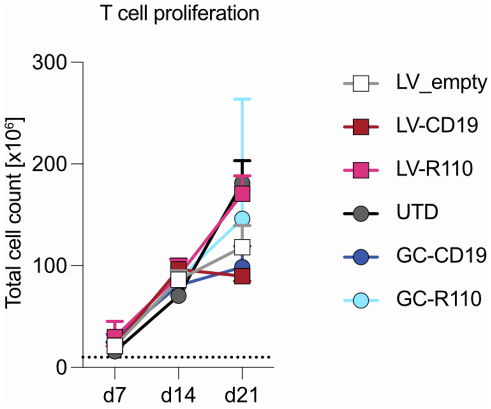

Figure S1. Proliferation of T cells in CAR expansion cultures over time. Proliferation quantified in HD-derived batches (n=3) and shown as mean ± SD. Dotted line indicates seeding amount.

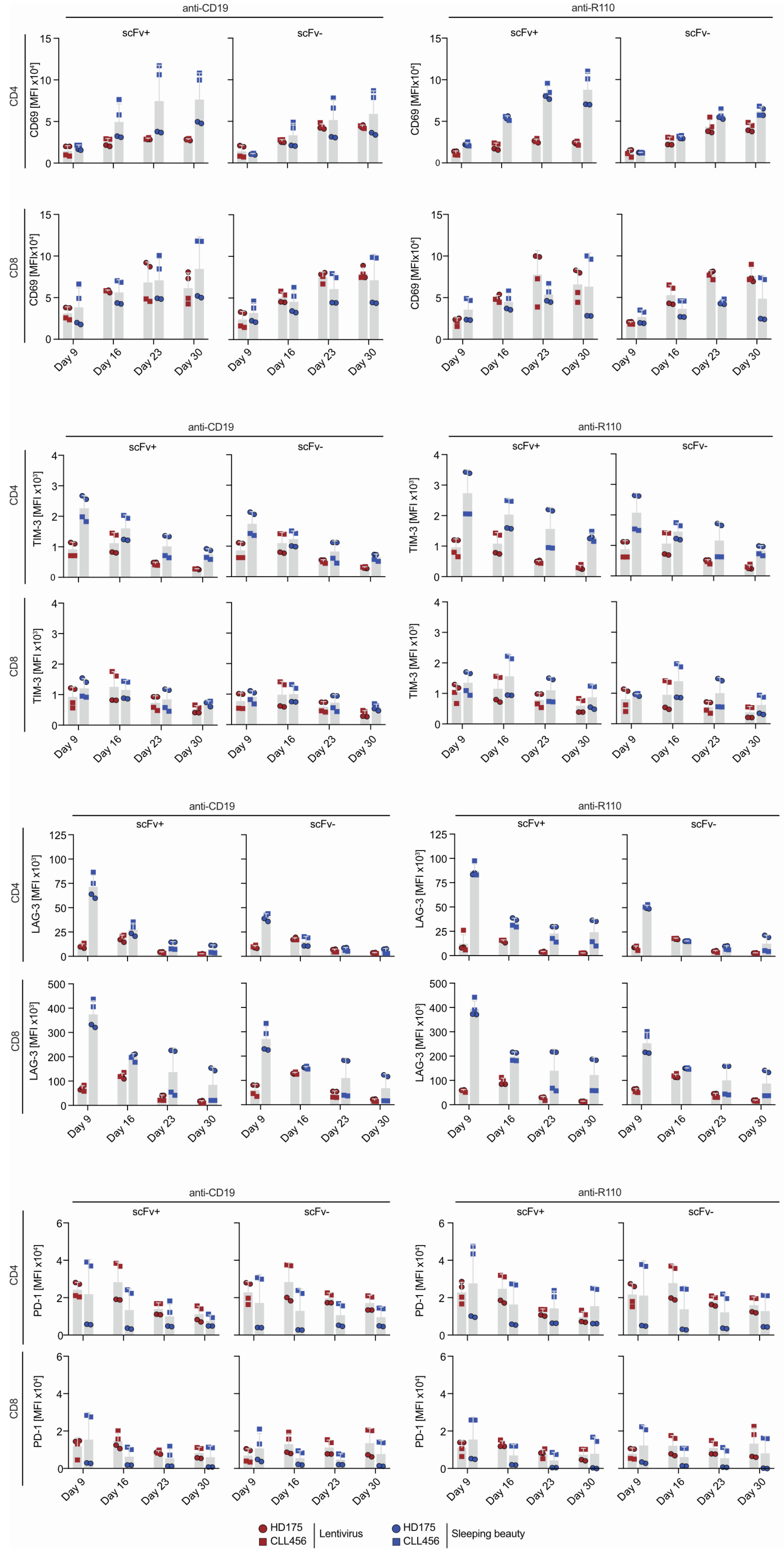
anti-CD19
anti-R110
scFv+
scFv-
CD4
CD8
CD69 [MFI x10⁴]
CD69 [MFIx10⁴]
TIM-3 [MFI x10³]
LAG-3 [MFI x10³]
PD-1 [MFI x10⁴]
Day 9
Day 16
Day 23
Day 30
HD175
CLL456
Lentivirus
HD175
CLL456
Sleeping beauty

Figure S2. Expression patterns of activation and co-inhibition markers in healthy donor and CLL patient-derived CAR T cells by multicolor flow cytometric analysis. (A) CD69, **(B)** TIM-3, **(C)** LAG-3 and **(D)** PD-1 mean fluorescence intensities (MFI) in CAR T cells separated by CD4, CD8, $scFv^+$ and $scFv^-$ expression. Bars represent the mean ± SD, each data point represents an individual donor.

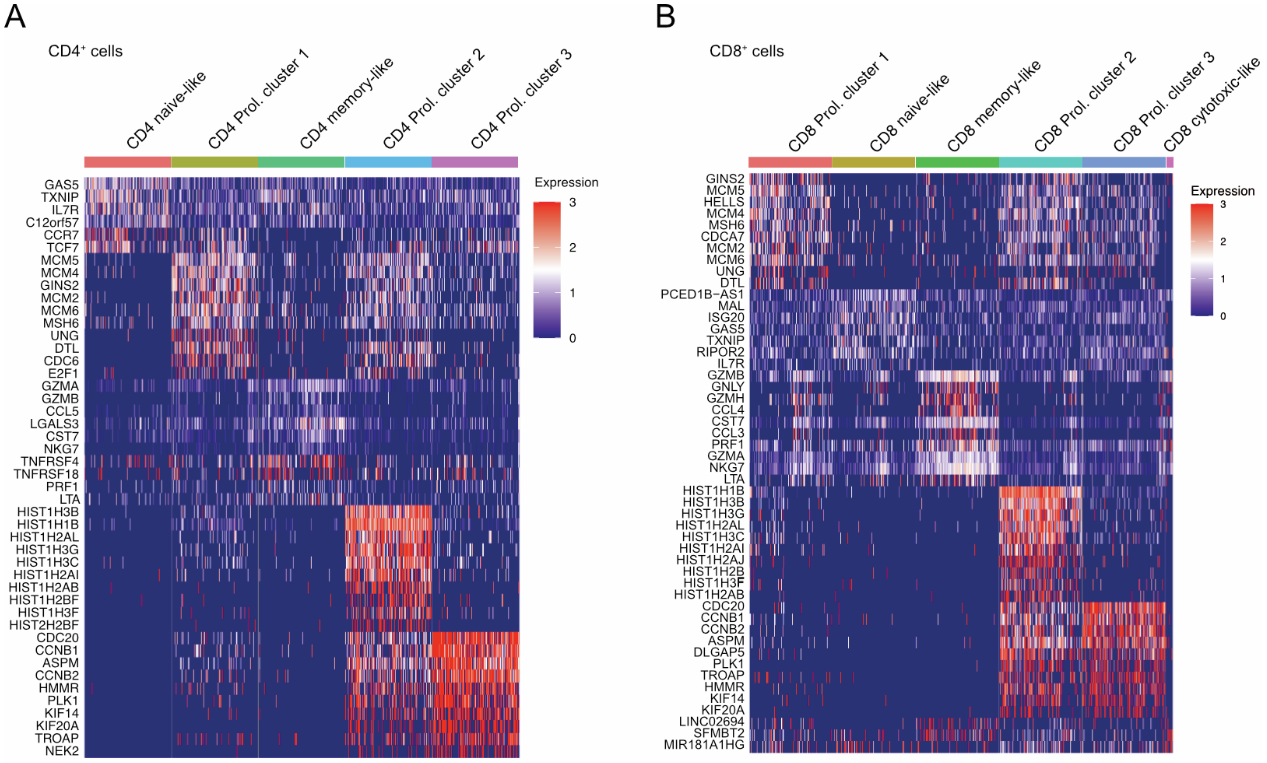

Figure S3. Top 10 differentially expressed genes per cluster in the CD4⁺ (A) and CD8⁺ (B) subsets.

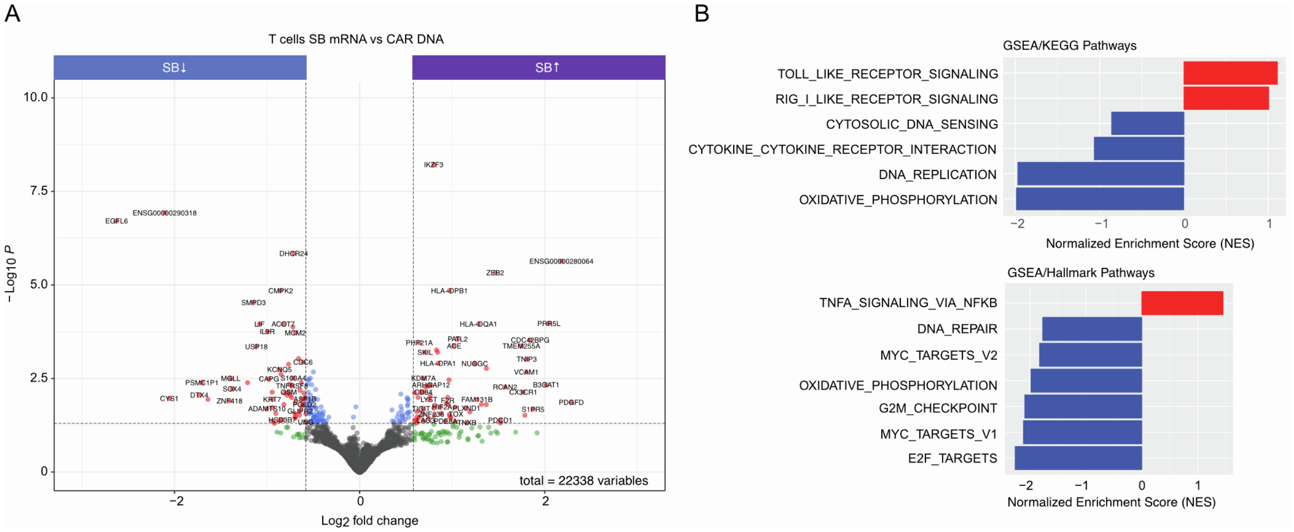

Figure S4. Characterization of transcriptomic signatures in T cells transfected with SB mRNA or CAR DNA. (A) Volcano plot showing genes differentially expressed genes (DEGs; adjusted $P < 0.01$) between T cells at day 14 post-transfection with either SB mRNA or CAR DNA alone. **(B)** Gene set enrichment of GSEA/KEGG (upper panel) and GSEA/Hallmark pathways (lower panel) for DEGs from (A).

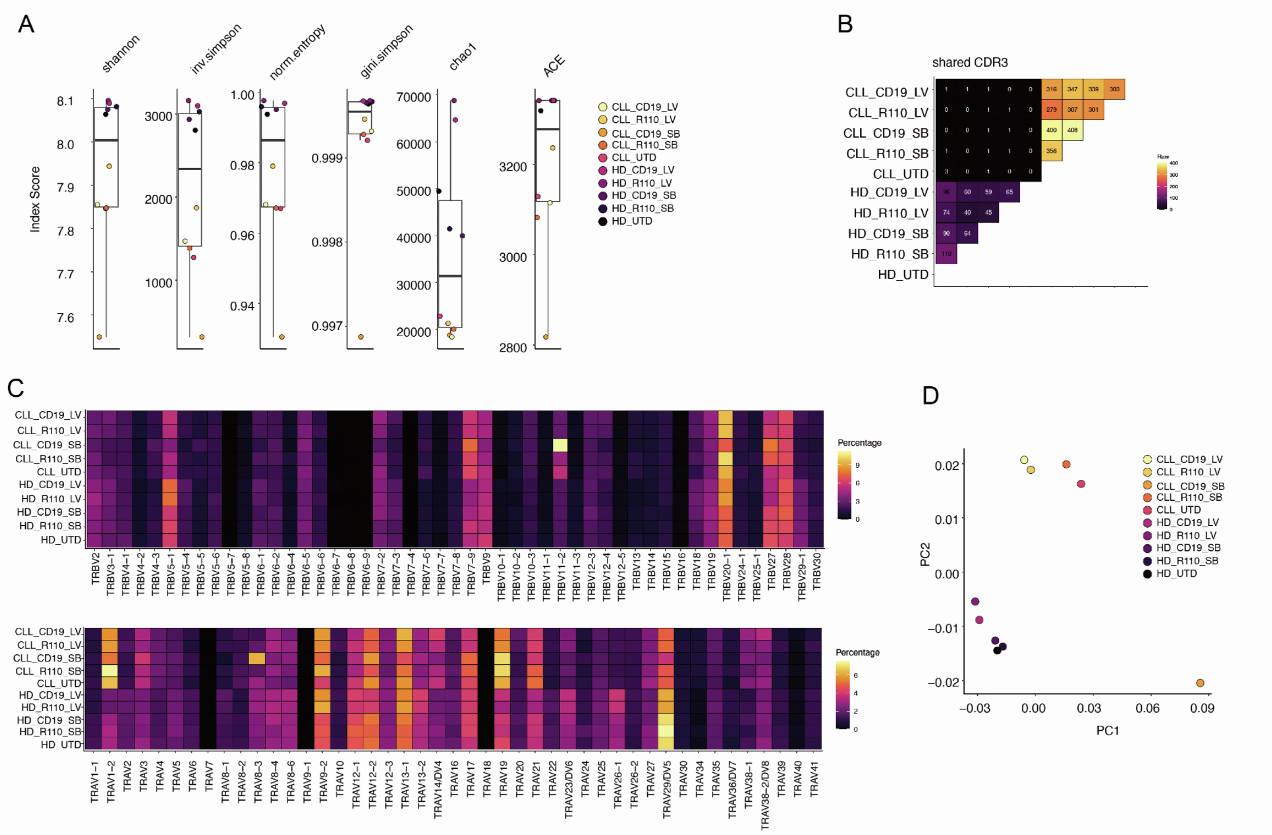

Figure S5. T cell receptor repertoire analysis of SB- and LV-manufactured CAR T cells. (A) Immune metrics. **(B)** Shared CDR3 sequences between donors and production batches. **(C)** Frequencies of TRBV gene usage. **(D)** PCA of TRBVJ usage.
