# Supplementary material for: CAR T cell engineering impacts antigen-independent activation and co-inhibition
Source: Mol Ther Methods Clin Dev. 2025 Sep 3;33(4):101586. doi: 10.1016/j.omtm.2025.101586 (PMC12464704; doi:10.1016/j.omtm.2025.101586)
Supplement: Document S2. Article plus supplemental information [file mmc2.pdf]

Molecular Therapy
Methods & Clinical Development
Original Article

CAR T cell engineering impacts antigen-independent activation and co-inhibition

Christoph Schultheiß,[1,2,7] Simon Stücheli,[1,2,7] Brenda Besemer,[1,2] Maja Kadel,[1,2] Paul Schmidt-Barbo,[1,2,6] Andreas Zingg,[1,3] Natascha Franz,[1,2] Sarah Adamo,[4,5] Frank Stenner,[1] Claudia Fischer,[1,2] Heinz Läubli,[1,3] and Mascha Binder[1,2,6]

[1]Division of Medical Oncology, University Hospital Basel, Petersgraben 4, 4031 Basel, Switzerland; [2]Laboratory of Translational Immuno-Oncology, Department of Biomedicine, University and University Hospital Basel, Hebelstrasse 20, 4031 Basel, Switzerland; [3]Laboratory of Cancer Immunotherapy, Department of Biomedicine, University and University Hospital Basel, Hebelstrasse 20, 4031 Basel, Switzerland; [4]Department of Medicine Huddinge, Center for Infectious Medicine, Karolinska Institutet, 14152 Stockholm, Sweden; [5]Department of Biomedicine, University and University Hospital Basel, Hebelstrasse 20, 4031 Basel, Switzerland; [6]Collaborative Research Institute Intelligent Oncology (CRIION), Georges-Koehler-Allee 302, 79110 Freiburg, Germany

Viral vectors have successfully modified T cells to express chimeric antigen receptors (CARs), leading to clinical approvals. However, their high cost and regulatory challenges hinder rapid clinical translation. Here, we demonstrate that our lentivirally (LV) manufactured R110-CAR T cells, targeting a leukemia neoepitope, can also be engineered using the non-viral sleeping beauty (SB) transposition with minimal-sized DNA vectors. Flow cytometry and single-cell sequencing were used to compare the two production modes using healthy donor and chronic lymphocytic leukemia (CLL)-patient-derived T cells and a CD19-CAR T cell control. SB products shifted toward CD8$^+$ subsets with activation/co-inhibition marker expression (CD69, LAG-3, and TIM-3) despite their naïve-like phenotype and lack of antigenic challenge. The CAR binding moiety modulated these patterns, with R110-CAR T cells showing more aberrant phenotypes. Moreover, SB engineering resulted in inflammatory signatures with RIG-I-like and TOLL-like nucleotide sensing potentially due to the transfection procedure. Patient-derived products showed fewer CAR-expressing cells, reduced proliferation clusters, and lower T cell diversity, particularly with SB manufacturing, indicating potential challenges with this method when engineering CLL T cells. Together, our data suggest that the engineering mode may substantially influence T cell properties and that these are further modulated by the CAR binding moiety and the T cell donor.

INTRODUCTION

Chimeric antigen receptor (CAR) T cells are powerful tools in oncology, offering a promising approach for targeted cancer therapy.[1,2] However, beyond the availability of tumor-specific target molecules, logistical complexity and high costs of manufacturing of autologous viral products limit CAR T cell availability.[3,4] Other manufacturing procedures such as sleeping beauty (SB) transposition of CAR genes may overcome some of these, especially financial, limitations, but such products are still experimental and no commercial SB-manufactured product has been licensed so far.[5–7]

Comparative studies on CAR T cell properties according to the mode of production are scarce, especially regarding their activation and co-inhibition profile. In the context of autologous CAR T cell therapy, where a patient's own often dysfunctional T cells are harvested, engineered, and reinfused, T cell inhibition is particularly problematic.[8] When these T cells are further subjected to the stress of genetic modification and expansion, their efficacy can be further diminished. This is exemplified by CAR T cells from healthy donors being better at controlling tumor growth in an *in vivo* model compared to CAR T cells derived from chronic lymphocytic leukemia (CLL) patients.[9] Moreover, certain single-chain variable fragments (scFvs) are associated with ligand-independent tonic signaling, which has been shown to reduce the efficacy of CAR T cells, thereby limiting their therapeutic potential.[10–12] This includes diminished cytokine production, reduced proliferative capacity, and impaired cytotoxic activity accompanied by the upregulation of inhibitory receptors such as PD-1, TIM-3, and LAG-3.[10,13] Whether the CAR-inherent level of tonic signaling may be modulated by the production mode remains unclear at this point.

In the work presented here, we show that our previously published lentivirally (LV) manufactured CAR T cells that are directed against the CLL neoepitope IGLV3-21^{R110} (named anti-R110)[14] can be engineered through non-viral SB transposition of CAR genes. This production method resulted in about equal efficacy of transgene expression, but the pattern of T cell differentiation, activation, and co-inhibition markers were substantially different and partially modulated by the specific CAR.

Received 14 March 2025; accepted 29 August 2025;
https://doi.org/10.1016/j.omtm.2025.101586.

[7]These authors contributed equally

Correspondence: Mascha Binder, MD, Division of Medical Oncology, University Hospital Basel, Petersgraben 4, 4031 Basel, Switzerland.
E-mail: mascha.binder@unibas.ch

© 2025 The Authors. Published by Elsevier Inc. on behalf of The American Society of Gene and Cell Therapy. This is an open access article under the CC BY license (http://creativecommons.org/licenses/by/4.0/).

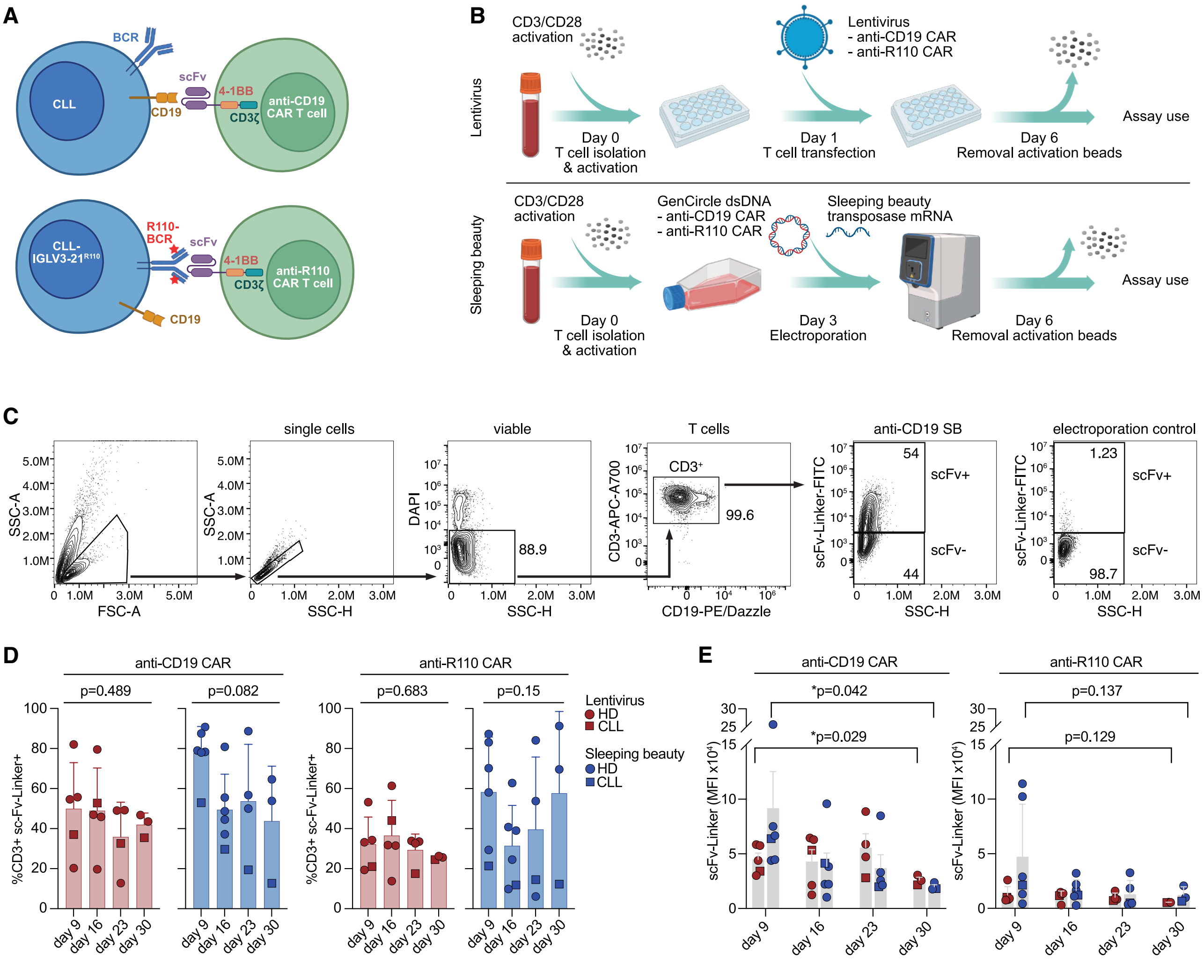

Figure 1. Development of a chimeric antigen receptor T cell targeting either CD19 or the IGLV3-21^{R110} neoepitope by LV transfection and SB transposition
(A) Schematic representation of the CD19 and IGLV3-21^{R110} (R110) CAR T targeting principle against chronic lymphocytic leukemia (CLL) cells. BCR, B cell receptor; scFv, single-chain variable fragment. (B) Schematic representation of the CAR T cell production workflow by LV transfection and SB transposition. (C) Gating strategy to determine CAR expression on T cells by flow cytometry. (D) Percentage of CAR-positive T cells determined by scFv expression using flow cytometry over the course of 4 weeks. Bars represent the mean ± SD; each data point represents an individual donor. Statistics: ordinary one-way ANOVA. (E) CAR density on T cells determined by the mean fluorescence intensity (MFI) of the scFv using flow cytometry over the course of 4 weeks. Bars represent the mean ± SD; each data point represents an individual donor. Statistics: two-sided t test between LV or SB samples from days 9 and 30.

RESULTS

Engineering and expansion of CD19- and R110-CAR T cells by LV transduction versus SB transposition

We used two constructs with potential applicability in CLL: an anti-CD19 CAR sequence containing 4-1BB and CD3ζ and a CAR sequence against the CLL neoantigen IGLV3-21^{R110} containing the same elements (Figure 1A). Both sequences were used to construct human T cells expressing the respective CAR either by LV transduction or by SB transposition (Figure 1B). We compared LV- and SB-manufactured CAR T cells derived from blood of healthy donor and CLL patient. Despite the cell damage caused by the electroporation procedure for the SB-engineered products, T cell expansion and cell viability recovered within the first week. Figure 1C shows the gating scheme for CAR-expressing T cells using the scFv-linker as detection marker. The CAR was expressed by approximately 40% of transduced or electroporated T cells 2 weeks from manipulation (Figure 1D). Notably, non-integrated CAR constructs contribute to CAR surface expression within the first 10–12 days in the SB approach before stable integration is achieved (Figure 1D). T cells proliferated at similar rates in the expansion culture independent of manufacturing approach (Figure S1). With SB transposition, we achieved somewhat lower percentages of CAR expression when CLL-derived T cells were used, potentially due to their greater fragility (Figure 1D). scFv membrane density was estimated based

on flow-cytometry-measured mean fluorescence intensity (MFI) of the CAR linker. After stable integration, scFv densities showed no consistent differences between SB- versus LV-manufactured CAR T cells (Figure 1E). R110-CAR expression was, however, consistently lower compared to CD19-CAR expression in both SB- and LV-manufactured products (Figure 1E).

CD4/CD8 distribution and differentiation stages of anti-CD19 and anti-R110 CAR T cells according to mode of production

Equal production media were used for LV and SB manufacturing. These contained interleukin-15 (IL-15) to maintain less differentiated subpopulations because these are believed to better persist in patients.[15,16] While LV manufacturing resulted in a $CD4^+$ dominant product, SB manufacturing showed a shift toward $CD8^+$ T cells upon expansion consistent with a previous study (Figures 2A and 2B).[17] The CD8 shift was more pronounced with anti-R110 CAR T cells. LV and SB production modes showed differences in T cell differentiation states of CAR products derived from the same T cell source (Figure 2C). While SB-manufactured products consisted essentially of a naïve-like T cell phenotype ($CD45RA^+$, $CCR7^+$), LV-manufactured CAR T cells showed a spectrum of $CD4^+$ differentiation stages including terminally differentiated cells ($CD45RA^+$, $CCR7^-$) (Figure 2C). Overall, $scFv^-$ T cells better retained the composition of T cell subtypes over time. Figure 2D shows integrated proportions of differentiation stages in T cells derived from healthy donors and a CLL patient.

Tonic CAR signaling and co-inhibition in SB- and LV-manufactured products

Interestingly, we observed that in the absence of target cells or antigen, $scFv^+$ $CD4^+$ T cells manufactured with SB showed an increase of the early activation marker CD69 compared to LV-generated CAR T cells as an indication of tonic CAR signaling (Figures 3A and S2). This was more consistently detected for the R110-CAR T cell product. CD69 also trended toward increased surface levels in $scFv^+$ $CD8^+$ cells, regardless of the manufacturing mode (Figures 3A and S2).

Next, we asked whether tonic CAR signaling leads to killing of bystander antigen-negative cells. We, therefore, calculated the ratio of specific killing by co-culturing different batches of R110- and CD19-CAR T cells with the antigen-positive NALM-6 cell line transduced with the LeGO-iZeo2 lentiviral vector encoding IGLV3-21^{R110} (NALM-6-R110) and the K562 cell line that is negative for CD19 and the R110 neoepitope (Figure 3B). R110-CAR T cells showed somewhat lower rates of specific killing with more background killing of antigen-negative cells compared to CD19 CAR T cells, but this was not associated with production mode, but rather correlated with effector-to-target (E:T) ratios (Figure 3C).

Next, we explored whether antigen-independent CD69 upregulation was accompanied by expression of other activation or co-inhibition markers. Therefore, we recorded expression of the classical T cell exhaustion markers PD-1, TIM-3, and LAG-3 in CAR T cells that were cultured without exposure to antigen or target cell line. Measurements were performed between day 9 and day 30 in order to follow dynamics over time. Different marker expression was observed depending on the construct and manufacturing mode as shown in Figures 3D and S2. While SB-manufactured products expressed slightly more TIM-3, especially on $CD4^+$ cells, there were more pronounced differences in PD-1 and LAG-3 expression between production modes. SB-manufactured T cells showed considerably lower PD-1 expression while exhibiting high levels of LAG-3 without antigen encounter. In contrast, LV-manufactured products showed higher PD-1 levels but low LAG-3. When visualizing marker expression by tSNE, LAG-3 expression was virtually restricted to $CD8^+$ subsets, while PD-1 was more expressed on $CD4^+$ cells and especially in the LV-manufactured products (Figure 3E). Overall, SB-manufactured T cells showed the highest LAG-3 expression in almost all $CD8^+$ cells while lacking PD-1 expression.

Single-cell RNA sequencing of SB- and LV-produced CAR T cells

To investigate the impact of CAR T cell manufacturing methods on the cellular states of engineered T cells, we performed integrated single-cell RNA and single-cell TCR sequencing on CD3-sorted CAR T cell cultures. These cultures included R110- and CD19-CAR T cells produced via SB and LV systems from both a healthy donor and a CLL patient. Untransduced T cells (UTDs) from SB production served as controls. Samples were collected on day 21 of production, yielding a total recovery of 67,799 cells (mean per sample 6,780; range 4,102–8,847) that passed quality thresholds, covering the major T cell subsets ($CD4^+$, $CD8^+$, and γδ T cells; Figures 4A–4C). Using standard surface markers and references from published single-cell atlases, we identified a core $CD4^+$ cluster with subsets displaying naive (*CD27*, *CCR7*, *SELL*, and *TCF7*), regulatory (*IL2RA*, *FOXP3*, and *RTKN2*), and memory-like (*ITGB1*) signatures (Figures 4A and 4B). A proliferation-associated cluster included both $CD4^+$ and naïve-like $CD8^+$ T cells (*MKI67* and *SOX4*). $CD4^+$ and $CD8^+$ T cells were further classified into central-memory-like and effector-memory-like subsets based on markers such as *CD27*, *GZMK*, *GZMB*, *KLRF1*, *CCR7*, *SELL*, and *LEF1* (Figures 4A and 4B). In addition, we identified a cluster of memory-like $CD4^+$ T cells with T regulatory cell (T_{reg})/effector-like features (*FOXP3*, *KLRB1*, and *ITGB1*), including cells expressing the MAIT cell marker gene *SLC4A10*. We also detected γδ T cells with both naïve-like and effector-like characteristics (*TRDC*, *TRGC1*, *GZMB*, and *GZMK*; Figures 4A and 4B). While all clusters were found with both CAR constructs and production modes, SB-manufactured T cells showed a trend toward expanded proliferative $CD4^+$/$CD8^+$ γδ T cells with high expression of *KLRF1* (proliferation cluster 2; Figures 4A and 4B). Additionally, γδ T cells and $CD8^+$ T cells were more prevalent in the SB product, while the LV product contained a higher proportion of $CD4^+$ T cells consistent with flow cytometry (Figure 4C).

Due to overlapping signatures in the overall $CD4^+$ and $CD8^+$ T cell data, we analyzed these subsets separately after reclustering (Figures 4D and 4E). Based on conventional T cell markers, cluster-specific differentially expressed genes, and published gene

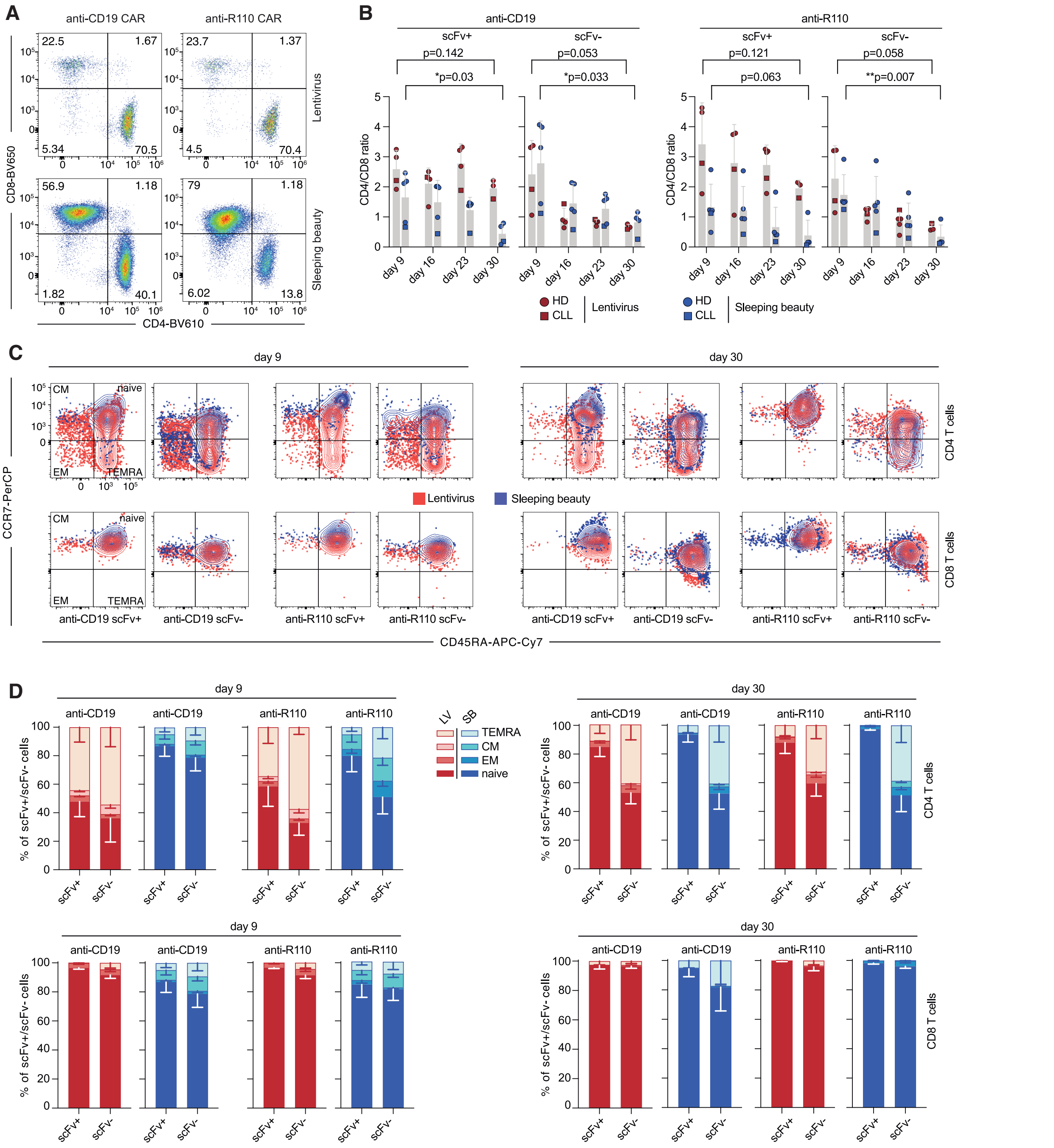

Figure 2. Characterization of the CD4 to CD8 ratio and T cell differentiation of CD19 and R110 targeting CAR T cells

(A) Representative image of the distribution of $CD4^+$ and $CD8^+$ CAR T cells on day 16. (B) Calculated $CD4^+$ to $CD8^+$ ratio of $scFv^{+/-}$ CAR T cells over the course of 4 weeks. Bars represent the mean ± SD; each data point represents an individual donor. Statistics: two-sided t test between LV or SB samples from days 9 and 30. (C) Representative T cell differentiation pattern of $scFv^{+/-}$ and $CD4^+/CD8^+$ CAR T cells derived from HD175 measured in technical duplicates by flow cytometry after 9 and 30 days. (D) Summarized results of the T cell differentiation pattern of pooled $scFv^{+/-}$ and $CD4^+/CD8^+$ CAR T cell samples of HD (n = 3) and CLL (n = 1), separated into $CD4^+$ and $CD8^+$ T cells and according to their scFv expression. Stacked bars represent the mean proportion ± SD.

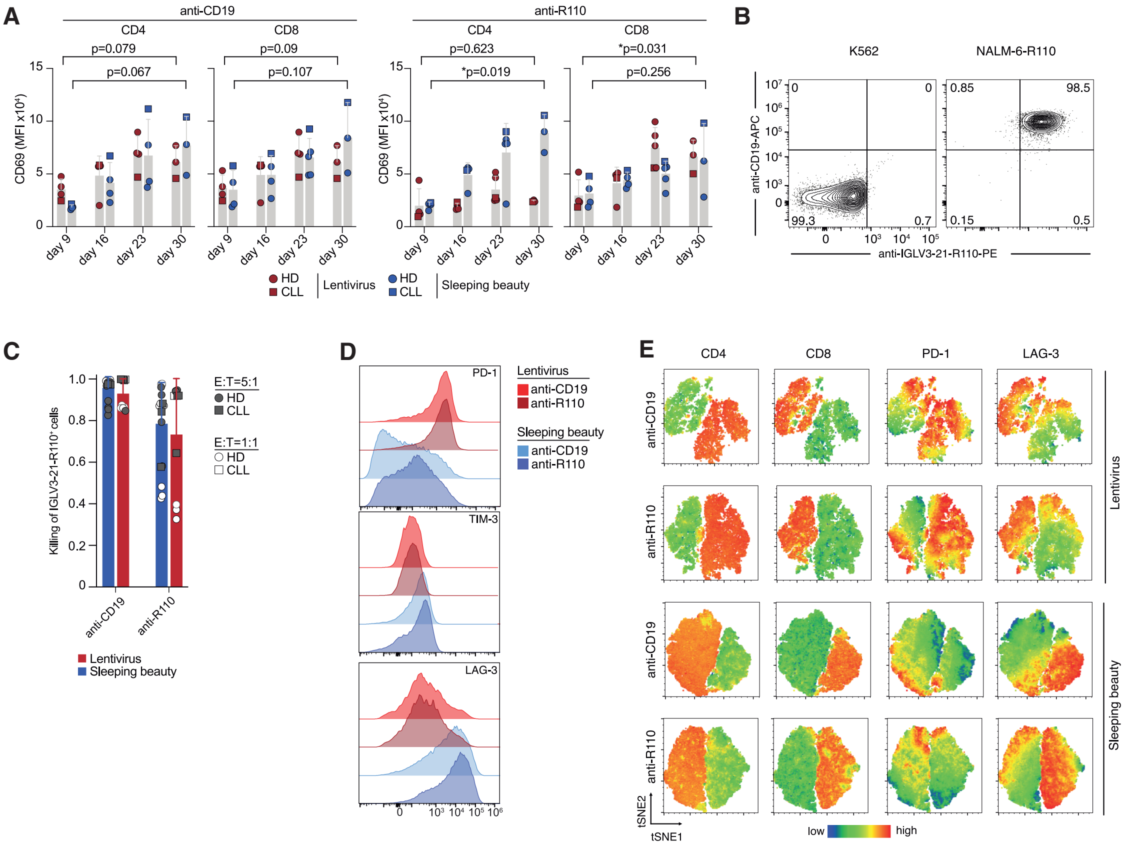

Figure 3. Expression patterns of activation and co-inhibition markers in healthy-donor- and CLL-patient-derived CAR T cells by flow cytometry

(A) CD69 surface expression in either scFv$^+$,CD4$^+$ or scFv$^+$,CD8$^+$ CAR T cells over the course of 4 weeks. Bars represent the mean ± SD; each data point represents an individual donor. Statistics: two-sided t test between LV or SB samples from days 9 and 30. (B) Representative flow cytometry image of target epitope expression in K562 and NALM-6-R110 cells. (C) Pooled results of *in vitro* co-culture assays indicating the killing of CD19$^+$, IGLV3-21^{R110+} NALM-6-R110 target cells in relation to CD19$^-$,IGLV3-21^{R110} K562 non-target cells. Bars represent the mean ± SD of pooled experiments. E:T ratios are indicated as symbol color and source donor as symbol shape. (D) Histogram of the PD-1, TIM-3, and LAG-3 expression in CD3$^+$ T cells (HD175) on day 9. (E) Visualization of the CD4, CD8, PD-1, and LAG-3 distribution pattern of CD3$^+$ T cells (HD175) on day 16. Technical duplicate measurements were concatenated and tSNE analysis performed with standard settings.

signatures for blood-derived T cells, we identified proliferating cells as well as naïve-like and memory-like subsets within both CD4$^+$ and CD8$^+$ populations (Figures 4D, 4E, S3A, and S3B). CAR-positive T cells expressing scFv were found across CD4$^+$ and CD8$^+$ clusters, particularly enriched in proliferating and naïve-like subsets, with fewer in memory-like subsets (Figures 4F and 4G).

In line with the detected CD45RA$^+$CCR7$^+$ surface patterns (Figure 2C), SB-manufactured CAR T cells exhibited slightly higher expression of markers associated with stem-cell-like memory T cells (T_{scm}; *FAS* (CD95), *IL2RB*, *CCR7*, *BCL2*) but did not display differences to LV-manufactured cells according to a global established T_{scm} signature (Figures 4H and 4I). CAR T cells, regardless of production method, showed limited effector memory (T_{em}) signatures and some central memory (T_{cm}) expression (Figure 4I). In both CD4$^+$ and CD8$^+$ CAR T cells, co-inhibition markers *LAG3* and *HAVCR2* (TIM-3) were considerably elevated compared to T cells not carrying the CAR (Figure 4J). SB-manufactured R110 CAR T cells showed more *LAG3* and *HAVCR2* transcripts compared to SB-manufactured CD19 CAR T cells (Figure 4K; mind different scales).

Pathway analysis in SB- and LV-produced CAR T cells

Next, we tested whether the SB and LV production modes resulted in activation or suppression of specific pathways. For this, we calculated differentially expressed genes (DEGs) in SB- and LV-manufactured products for the CD4$^+$ scFv$^+$ and CD8$^+$ scFv$^+$ populations as well as the scFv$^-$ bystander cells (Figure 5). Interestingly, across all DEG analyses, scFv$^+$ cells consistently yielded higher numbers of DEGs (Figures 5A and 5D). Independent of scFv expression, CD4$^+$ T cells from the SB batch were characterized by increased expression of the chemokine *CCL5* (RANTES), the proliferative transcription factor *HDGLF3*, and of cytotoxicity markers (*GZMA* and *GZMB*) including the transcription factor *BHLHE40* that is crucial for regulating T cell effector functions (Figure 5A).[18] Gene set enrichment analysis (GSEA) revealed that T cells from the SB production had

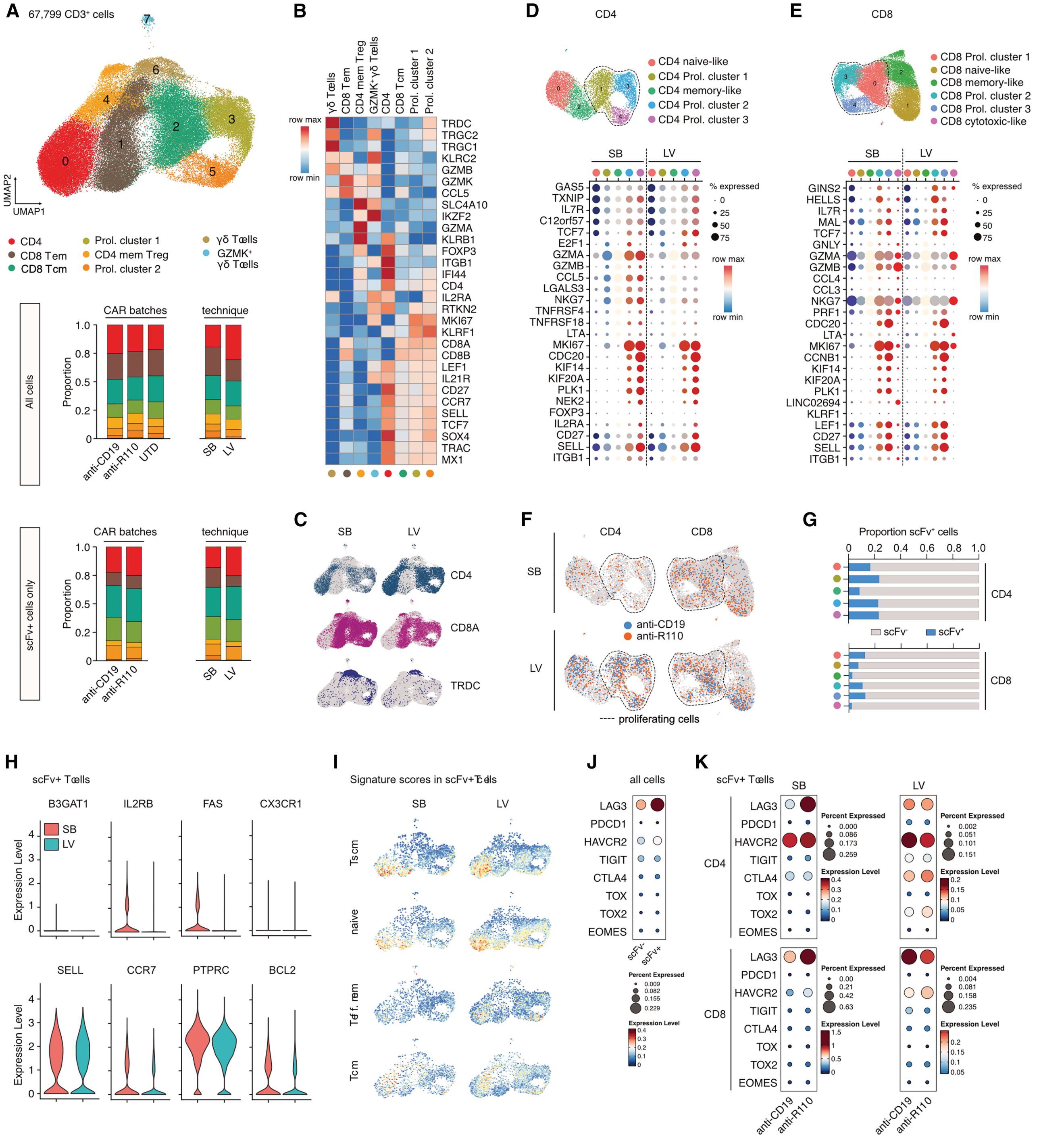

Figure 4. Single-cell RNA sequencing analysis of $CD3^+$ T cells from SB and LV production pools

(A) Uniform manifold and approximation projection (UMAP) plot of $CD3^+$ T cells from the SB and LV production pools. Stacked bar plots display the cluster cell distribution based on CAR T production batches (including UTD control cells) or production mode (SB, sleeping beauty transposition; LV, lentiviral transduction) for all $CD3^+$ T cells and CAR T cells only ($scFv^+$ cells). (B) Heatmap of average normalized expression for conventional T cell marker genes in each cluster. (C) UMAP plot showing *CD4*, *CD8A*, and *TRDC* expression in $CD3^+$ T cells with respect to CAR T production mode. (D) UMAP plot of $CD4^+$ T cells after re-clustering. Expression of T cell markers and selected cluster-defining genes as dot plot grouped by production mode. Dotted lines indicate proliferating T cell clusters. (E) UMAP plot of $CD8^+$ T cells after re-clustering. Expression of T cell markers and selected cluster-defining genes as dot plot grouped by production mode. Dotted lines indicate proliferating T cell clusters. (F) UMAP plot of $CD4^+$ and $CD8^+$ T cell

(legend continued on next page)

higher expression of proinflammatory genes related to tumor necrosis factor (TNF), interferon (IFN), and JAK-STAT signaling (Figure 5B). In contrast, SB-manufactured T cells displayed decreased levels of genes for cell-cycle control, replication, and proliferation (*MYB*, *PCLAF*, *H2AFZ*, and *HIST1H1A*) (Figures 5A and 5B). The scFv$^+$ CD4$^+$ T cells from the SB production were especially characterized by the enrichment of genes (*DDX60*, *DDX3X*, and *TBK1*) that act in the RNA-/DNA-sensing RIG-I and TLR signaling pathways including the pivotal mediators and effectors from the nuclear factor κB (NF-κB) (*IKBKE*, *TRAF2*, and *RELA*) and IFN signaling pathways (*IRF3*, *IRF7*, *IFIH1*, and *ISG15*) (Figures 5B and 5C). In addition, CD4$^+$ T cells from the SB batches also had high levels of the E3 ISGylation ligase 5 (*HERC5*) that mediates activating ISGylation of the cytosolic DNA sensor STING (Figure 5A).[19] Notably, we did not observe enriched RIG-I and TLR gene signatures in CD8$^+$ CAR T cells from the SB production (Figures 5D and 5E). Instead, CD8$^+$ scFv$^+$ T cells were characterized by increased expression of *CCL3*, *CCL4*, and the FYN/CSK/Abl-interacting transmembrane adaptor protein *PAG1* that negatively regulates T cell activation and is linked to anergy (Figure 5D).[20,21] Moreover, CD8$^+$ scFv$^+$ cells displayed enriched p53 pathway signatures, which may be associated with CD8$^+$ T cell exhaustion,[22] and had increased levels of *LAG3* as compared to cells from the LV production (Figure 5D). CD8$^+$ CAR T cells from the SB production shared the general enrichment of pro-inflammatory signatures with their CD4$^+$ counterparts (Figures 5D and 5E). To validate the observed RIG-I/TLR signatures and determine whether they are induced by RNA or DNA in the transfection process, we electroporated CD3$^+$ T cells with either the SB mRNA or the CAR DNA constructs alone. RNA sequencing analysis between both conditions confirmed the increased transcript levels seen with single-cell RNA sequencing (scRNA-seq), including genes such *PDCD1*, *LAG3*, *TIGIT*, and *TOX*, and revealed similar pathway enrichment patterns (Figure S4). In addition, flow cytometry analysis conducted 7 days post-transfection showed a substantial increase in surface expression of CD69, PD-1, TIM-3, and LAG-3, particularly after mRNA delivery (Figure 5F).

Donor cell constitution impacts transcriptomic signatures of CAR T cells

To test for donor-dependent transcriptome differences, we separated the single-cell dataset into HD- and CLL-derived cells (Figures 6A and 6C). While all defined T cell subpopulations (Figure 4) encompassed cells from both donors (Figures 6A and 6B), the CLL-derived T cell pools had lower numbers of proliferating cells, but higher numbers of γδ, CD4$^+$, CD8$^+$ T_{em}, and CD4$^+$ memory T cells (Figures 6A and 6B). CLL-derived products had generally increased transcript levels of *LAG3*, *CD69*, *ID2*, and *SLAMF7*, several chemokines and long non-coding RNAs (lncRNAs), as well as HLA class molecules and cytotoxicity markers (Figures 6A and 6C). In contrast, genes related to cell-cycle control and proliferation exhibited pervasively lower transcript levels as compared to HD-derived T cells (Figures 6A and 6C). Within the reclustered CD4$^+$ and CD8$^+$ subsets (Figures 4D and 4E), we detected an enrichment of memory-like cells in CLL-derived T cells independent of production mode, while HD-derived T cells were more enriched for proliferating cells (Figure 6D).

Finally, we tested for donor-dependent differences in activation/co-inhibition markers. As compared to HD-derived cells, the SB-associated increase of *LAG3* expression was more pronounced in CD4$^+$ than in CD8$^+$ scFv$^+$ T cells derived from CLL patients (Figure 6E). In addition, we observed higher levels of *PDCD1*, *TIGIT*, and *TOX2* in CLL-derived CD4$^+$ T cells with scFv expression from the SB production (Figure 6E). CAR T cells from the LV production had lower levels of these markers and showed no clear donor-dependent differences (Figure 6E).

TCR repertoire analysis of CAR T cells

Single-cell TCR analysis demonstrated polyclonality across all products, with no notable differences between CAR T cells manufactured via SB and LV methods. T cell characteristics, however, varied significantly with the donor source. CAR T products derived from the CLL donor exhibited lower T cell richness and diversity compared to those from the HD, highlighting a restriction in the CLL donor's T cell repertoire (Figure S5A). This limitation was further confirmed by TCR repertoire overlap analysis, which revealed substantial clonal overlap between the CLL donor's CAR T products, in contrast to the HD-derived products, which showed minimal clonal overlap (Figure S5B). TRA and TRB gene analyses identified a broad spectrum of gene usage in both donors, with some patterns specific to each donor (Figure S5C). This trend was reflected in a principal-component analysis (PCA) of TRBVJ gene usage, where CAR T cell products from each donor clustered together. No distinct differences were observed based on production method or CAR binding moiety (Figure S5D).

DISCUSSION

T cell dysfunction is one of the major roadblocks in the development and application of CAR T cell therapies.[8] There is only scarce data on how the production mode and the binding moiety may impact CAR T cell phenotypes. Here, we provide a head-to-head comparison of different production modes—lentiviral transfection and sleeping beauty transposition—of R110- and CD19-directed CAR T cells with a focus on T cell activation and co-inhibition marker expression.

In our study, we demonstrate that different CAR constructs exhibit distinct patterns of marker expression for T cell activation and

subsets with marked scFv$^+$ T cells per production mode. (G) Proportion of scFv$^+$ cells per cluster in (F). (H) Violin plots showing expression of marker genes for stem cell-like T memory cells (T_{scm}) in scFv$^+$ cells from the SB and LV production. (I) UMAP plot showing enrichment of gene signature scores for the indicated T cell subpopulations depending on production mode. (J) Dot plot showing the expression of selected T cell exhaustion/activation markers in scFv$^{+/-}$ cells. (K) Dot plot showing the expression of selected T cell exhaustion/activation markers in scFv$^+$ CD4$^+$ or CD8$^+$ T cells depending on production mode.

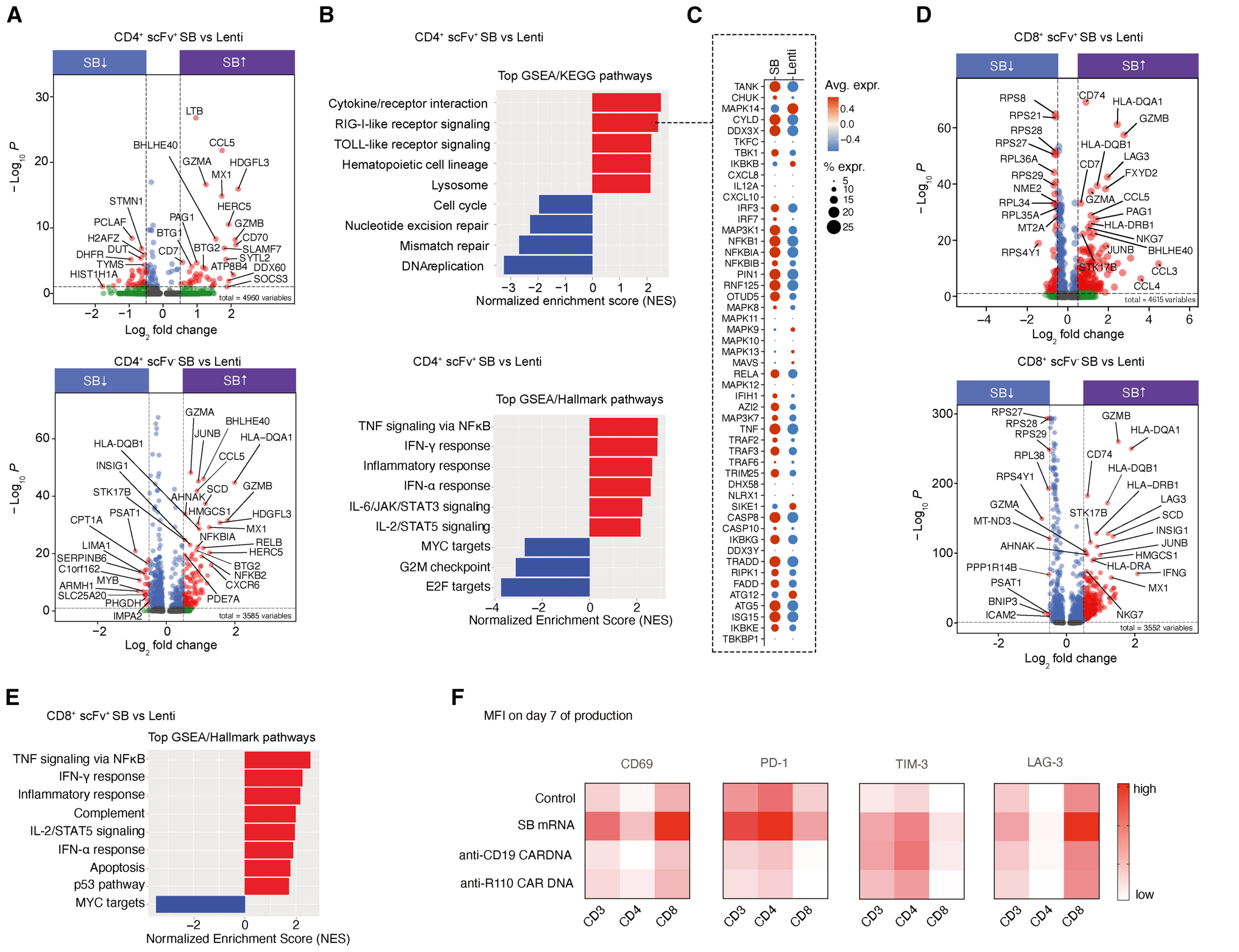

Figure 5. Characterization of production-protocol-specific transcriptomic signatures in CD3$^+$ T cells from SB and LV production pools

(A) Volcano plot showing genes differentially expressed (adjusted $p < 0.01$) between SB and LV production modes for CD4$^+$ scFv$^+$ (upper panel) and CD4$^+$ scFv$^-$ T cells (lower panel). (B) Gene set enrichment of GSEA/KEGG (upper panel) and GSEA/Hallmark pathways (lower panel) in SB-manufactured scFv$^+$ CD4$^+$ T cells as compared to the LV production. (C) Dot plot showing the average normalized expression of genes from the RIG-I receptor signaling gene set in SB- and LV-produced scFv$^+$ CD4$^+$ T cells. (D) Volcano plot showing genes differentially expressed (adjusted $p < 0.01$) between SB and LV production modes for CD8$^+$ scFv$^+$ (upper panel) and CD8$^+$ scFv$^-$ T cells (lower panel). (E) Enrichment of GSEA/Hallmark pathway genes in SB-manufactured scFv$^+$ CD8$^+$ T cells as compared to the LV production. (F) Heatmap displaying surface expression (MFI) of indicated markers in bulk CD3$^+$ or CD4$^+$ and CD8$^+$ T cells as determined by flow cytometry on day 7 for mono-electroporation of SB mRNA, anti-CD19, or anti-R110 CAR DNA constructs. Control T cells underwent electroporation only.

co-inhibition. Notably, we observed substantial differences between SB and LV production methods, even when using identical CAR sequences, T cell donors, and culture conditions—factors known to critically impact the final CAR T cell product.[15] Our data suggest that especially the delivery of SB mRNA is a dominant driver of the exhausted-like phenotypes by eliciting intracellular nucleic-acid-sensing pathways like RIG-I. Interestingly, upregulation of RIG-I signaling has been reported in terminally exhausted CD8$^+$ T cells and was linked to reduced CD8$^+$ T cell effector functions in tumor-infiltrating lymphocytes.[23] Also, other product-defining features were significantly affected by the production mode, such as CD4/8 ratio, differentiation stage, or CD69 expression as a marker for antigen-independent tonic activation. While no differences in *in vitro* killing capacities were observed between the two manufacturing approaches within the 4-week post-production period, the activation-/exhaustion-like patterns detected in the SB process may predispose patient-derived T cells to a faster decline in effector functions when exposed to high antigen loads *in vivo*. This is especially relevant in CLL, which is known for its particular complex and dysfunctional T cell landscape.[24–28] Our patient-derived data align to this pattern but clearly require further validation as they are based on a single patient. These findings are crucial as they suggest that the optimization of CAR constructs extends

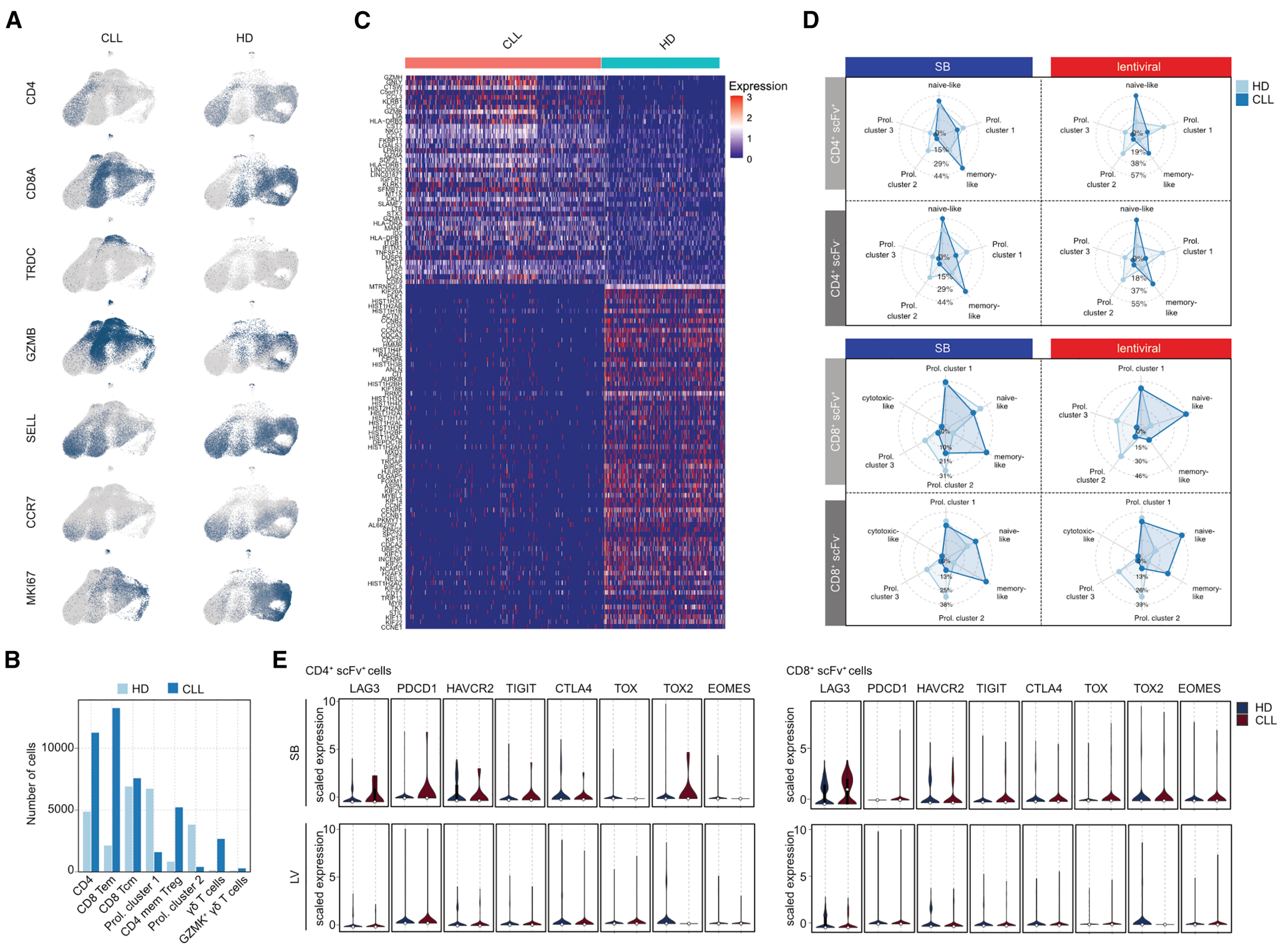

Figure 6. Analysis of donor-dependent impact on SB- and LV-manufactured CAR T cell transcriptomes

(A) UMAP plot of healthy donor (HD)- or CLL-patient-derived $CD3^+$ T cells displaying expression of selected T cell markers. (B) Cell numbers in the indicated subpopulations (Figure 4) depending on the donor. (C) Heatmap showing differential expression of genes between HD-/CLL-derived $CD3^+$ T cells. (D) Radar plot displaying the contribution of HD-/CLL-derived $CD4^+$ scFv $^\pm$ and $CD8^+$ scFv $^\pm$ T cells to the single subsets in the bulk $CD4^+$ and $CD8^+$ populations from the SB and LV production pools. (E) Violin plot showing expression of indicated exhaustion/co-inhibition markers in HD-/CLL-derived $CD4^+$ and $CD8^+$ $scFv^+$ T cells.

beyond the design of co-stimulatory domains or the configuration of the binding moiety, supporting that each construct must also be rigorously tested under various manufacturing conditions.

In a related approach, a previous study compared the phenotypes of CD19-targeting CAR T cells either manufactured using electroporation and the PiggyBac transposase system or produced by lentiviral transduction.[29] The authors noted higher CAR density and cytokine release in CAR T cells generated with the transposase system but no significant differences regarding CD69 or PD-1 surface expression or anti-tumor efficacy in a murine xenograft model.[29] CAR T cells produced with the transposase system displayed lower frequencies of T_{cm} and T_{em} phenotypes as their lentiviral counterparts and had higher expression of genes related to immunological and inflammatory pathways as shown in our study.[29] However, their analyses were restricted to the first week after CAR T production.[29]

There are some data linking CAR T cell phenotypes to efficacy outcomes of CAR T cells in patients. While some studies suggested that a balanced ratio of CD4 and CD8 cells in the product would support persistence and eradication of the malignant clone in patients,[30,31] others did not support this conclusion.[9] Moreover, in one study, early memory T cell differentiation in the CAR T cell product was found to correlate with better responses while an effector or exhausted T cell differentiation was not.[9] In another study, peak CAR T cell detection in patients correlated with the percentage of a naive CAR^+ $CD8^+CD45RA^+CCR7^+$ T cell subset in the CAR T cell product.[32] Furthermore, the percentage of $PD\text{-}1^+CD8^+$ CAR T cells co-expressing either TIM-3 or LAG-3 correlated with a lower response to therapy.[9] CD62L (*SELL*), also known as L-selectin, is another marker of naive T cells, including stem cell memory T cells, but also central memory T cells. Its enhanced expression was shown to lead to better control of tumor growth in an *in vivo*

model, and its expression level was associated with a better *ex vivo* expansion.[33–35] Overall, T cell subset distribution and co-inhibitory receptor expression as well as prior antigen encounter influence outcomes with CAR T cells,[15,35,36] suggesting that the differences associated with production mode and CAR specificity seen in our study may be of clinical relevance.

The determinants of T cell dysfunction in the context of CAR T cells remain poorly defined. T cell exhaustion is considered the result of continuous exposure to antigen[8,37] but may also occur without antigen exposure in the case of CAR T cells, e.g., by self-aggregation of the CAR resulting in tonic signaling.[8] It has been assumed that tonic signaling could not only be a feature of the CAR sequence itself but also a function of CAR membrane density. To this end, "self-driving" armored CAR T cells that upregulate CAR expression only in case of antigen encounter were developed and tested preclinically.[38] As opposed to this assumption, we found that the pattern of high LAG-3 (and also TIM-3) expression was not associated with higher membrane expression of CAR molecules. In contrast, we observed higher basal LAG-3 expression in SB-manufactured R110-CAR T cells that showed much lower CAR density than CD19-directed CAR T cells. This suggests that, at least in our model, high CAR expression is not a critical inducer of exhaustion-associated surface molecules LAG-3 and TIM-3 by tonic CAR signaling.

We observed a low percentage of scFv$^+$ T cells and a depletion of proliferative T cell clusters in CAR T cells derived from the CLL donor. These effects were more pronounced in SB-manufactured products, suggesting that the stress associated with SB transposition may further impact the already fragile T cells from patients, thereby impairing their proliferative capacity. In conditions like CLL, where T cell defects are well documented, it will be essential to investigate this further to optimize transgene delivery (e.g., reduction of transfected SB mRNA amounts) and achieve a cell product that can proliferate and persist effectively in patients.

In summary, while CAR T cells hold great promise in oncology, T cell functionality remains a critical challenge, particularly in the autologous setting. Addressing this issue is essential for enhancing the efficacy and expanding the applicability of CAR T cell therapies across a broader range of cancers.

MATERIALS AND METHODS

Cell lines and CLL patient and healthy donor blood cells

HEK293T, K562, and NALM-6 cells were purchased from the DSMZ (German Collection of Microorganisms and Cell Cultures GmbH). For ectopic expression of the IGLV3-21^{R110} light chain in NALM-6 cells (NALM-6-R110), the coding sequence was cloned into the lentiviral gene ontology (LeGO) vector LeGO-iZeo2, and positively transfected cells were selected by culturing cells in the presence of 0.3 mg/mL zeocin (#ant-zn-1, InvivoGen, Toulouse, France). NALM-6 and NALM-6-R110 cells were maintained in RPMI-1640 (#61870, Thermo Fisher Scientific, Waltham, MA, USA), K562 in IMDM (#I3390, Sigma-Aldrich, St. Louis, MO, USA), and HEK293T in DMEM (#31966-021, Thermo Fisher Scientific) medium. All three media were supplemented with 10% heat-inactivated fetal bovine serum, 100 U/mL penicillin, and 100 μg/mL streptomycin.

Blood samples from CLL patients were collected after informed consent as approved by the ethics committees of Halle-Wittenberg. Peripheral mononuclear cells (PBMCs) from CLL patients or healthy donors were isolated by Ficoll gradient centrifugation and primary T cells isolated using the Pan T cell Isolation Kit (#130-096-535, Miltenyi Biotec, Bergisch Gladbach, Germany).

CAR constructs

The anti-CD19 CAR construct was derived from the FMC63 antibody and contains the scFv in VL-VH configuration.[39] The scFv sequence for the anti-IGLV3-21-R110 construct is a humanized derivative of the murine anti-IGLV3-21R110 antibody from AVA Lifescience GmbH (Denzlingen, Germany), as previously described.[14] Both scFvs are cloned in the same second-generation backbone containing 4-1BB and CD3ζ costimulatory domains and a truncated epidermal growth factor receptor (tEGFR) for monitoring.[39] For SB production, CARs were cloned to into GenCircle minimal-sized plasmids with an EFS promoter, bGH polyadenylation site, and two delimiting ITR recombination sites. GenCircle is a proprietary technology of GenScript (Piscataway, NJ, USA).

Lentiviral production

Lentiviral particles were generated by transient transfection of HEK293T cells using the third-generation packaging plasmids pMDLg/pRR3 and pRSV-Rev and the VSV-G envelope protein as described earlier.[40] Specifically, five million HEK293T cells were seeded per 10 cm dish. After 24 h, the medium was replaced with fresh medium containing 25 μM chloroquine. The cells were then transfected with the lentiviral vector containing the CAR sequence of interest along with lentiviral packaging plasmids using the calcium phosphate transfection method.[14] Five hours post-transfection, the medium was changed, and the cells were incubated for 3 days. Subsequently, the supernatant was collected, filtered (0.2 μm), mixed with a 40% w/v PEG-8000 solution (25% PEG solution, 75% supernatant), and stored at 4°C overnight. The mixture was centrifuged (4°C, 45 min, 1,500 rcf), the supernatant discarded, and the viral particles resuspended in activation medium (RPMI-1640, 10% heat-inactivated human serum, 100 U/mL penicillin, 100 μg/mL streptomycin, 50 μM 2-mercaptoethanol, 25 IU/mL IL-2 [#78036.1, StemCell Technologies, Vancouver, BC, Canada], 10 ng/mL IL-15 [#200-15-50UG, PeproTech, Thermo Fisher Scientific]). The viral particles were either immediately used for T cell transfection or stored at −80°C.

Preparation of CAR T cells by lentiviral transduction or sleeping beauty transposition

For lentiviral transduction, T cells were activated using CD3/CD28 Dynabeads (#11132D, Thermo Fisher Scientific) in a

24-well format (1×10^6 cells per well, bead-to-cell ratio 1:1) in activation medium. After 24 h, 66% of the medium was replaced with fresh activation medium containing 0.5 mg/mL Synperonic-F108 and lentivirus encoding the respective CAR constructs. Following an additional 24 h, 50% of the medium was replaced with fresh activation medium. After another 48 h, the T cells were transferred to PRIME XV T cell CDM (91154-1L, FUJIFILM Irvine Scientific, Santa Ana, CA, USA) containing 10 ng/mL IL-15 and placed in a G-Rex-24-well format (#80192M, Wilson Wolf, St. Paul, MN, USA). On day 6 (144 h post-T cell seeding), the CD3/CD28 beads were removed, and the medium was replaced.

For CAR T cell production using the SB transposase system, T cells were similarly activated using CD3/CD28 Dynabeads (bead-to-cell ratio 1:1) in activation medium. After 72 h, 10×10^6 T cells were electroporated using the MaxCyte GT Electroporation System (MaxCyte, Rockville, MD, USA) following its T cell Protocol 3. The electroporation was performed in the presence of 40 μg/mL SB transposase mRNA (custom-made by Etherna [Niel, Belgium] with CleanCap [TriLink, San Diego, CA, USA] and a plasmid-encoded polyA tail 94A) and 150 μg/mL GenCircle CAR vector construct (GenScript, Piscataway, NJ, USA) in PRIME XV T cell CDM, using R-50 × 3 cuvettes (#ER050U3-10, MaxCyte). Electroporated T cells were immediately transferred to a G-Rex-24-well format, and after 4 hours, IL-15 was added to achieve a final concentration of 10 ng/mL. On day 6 (144 h post-T cell seeding), the CD3/CD28 beads were removed. All CAR T cells used in this study were generated from freshly isolated T cells and cultured at 37°C in a 5% CO_2 atmosphere in PRIME XV T cell CDM with 10 ng/mL IL-15. The medium was refreshed once per week. Viable T cell counts in expansion cultures were quantified by trypan blue staining using the Cell Viability Analyzer Vi-Cell XR (Beckman Coulter).

In vitro co-culture assays

Target cells seeded at 3×10^4 cells/well in a 96-well plate were co-incubated with effector cells at an E:T ratio of 5:1 or 1:1 in standard RPMI-1640 media (10% heat-inactivated fetal bovine serum, 100 U/mL penicillin, 100 μg/mL streptomycin). Total numbers of CAR T cells were adjusted based on scFv$^+$ expression as determined by flow cytometry. Immediately after combining target and effector cells, half of each well was subjected to flow cytometric analysis and the other half incubated for 24 h.

Killing of target cells was assessed by gating for viable (DAPI-negative, #130-111-570, Miltenyi Biotec) CD3$^-$,CD19$^+$,R110$^+$ target NALM-6-R110 cells and by normalizing their percentage after 24-h incubation to 0-h incubation time. The same normalization was applied to DAPI$^-$CD3$^-$CD19$^-$R110$^-$ non-target K562 cells. Furthermore, the resulting values were used for an additional normalization step (Equation 1).

$$1 - \left(\frac{\left[\frac{\%\ target\ at\ 24h}{\%\ target\ at\ 0h} \right]}{\left[\frac{\%\ non-target\ at\ 24h}{\%\ non-target\ at\ 0h} \right]} \right) \qquad \text{(Equation 1)}$$

Flow cytometry was performed by washing the cells with PBS, followed by an incubation with primary unlabeled antibodies in staining buffer (1% w/v bovine serum albumin in PBS) for 30 min at 4°C in the dark. Cells were washed with PBS, incubated with labeled secondary antibodies and labeled primary antibodies in staining buffer for 30 min at 4°C in the dark, washed by PBS, resuspended in DAPI (1:200 dilution), and measured on a CytoFLEX S (Beckman Coulter, Brea, CA, USA). T-distributed stochastic neighbor embedding (tSNE)[41] analysis was performed by concatenating the duplicate measurements of the samples and using the embedded tSNE function of FlowJo (v10.10) with standard settings after gating for single DAPI$^-$,CD3$^+$ cells. Antibodies used for flow cytometry were diluted at 1:200 unless otherwise specified and listed in Table S1.

Single-cell RNA and VDJ sequencing

Single-cell RNA and T cell receptor (TCR) VDJ sequencing was performed using the Chromium Next GEM Single Cell 5′ Kit v2, Human TCR Amplification, and Human TCR Amplification kits (10× Genomics, Pleasanton, CA, United States) as described previously.[42,43] For library preparation, live (DAPI-negative) T cells from the SB and LV expansion cultures were FACS-sorted after staining the cells at 4°C for 30 min using an FITC-labeled anti-CD3 (clone SK7, #345763, BD BioSciences, Franklin Lakes, NJ, USA) antibody. Quality control steps were performed using an Agilent TapeStation (Agilent Technologies, Inc., Santa Clara, CA, United States). Sequencing was done on an Illumina NovaSeq 6000 system (Illumina, San Diego, CA, United States). Raw gene expression and TCR data were processed to read counts with the Cell Ranger pipeline (v7.0.1) (10× Genomics), which includes alignment to the human reference genome (GRch38), filtering, and barcode counting. Processed read count matrices were analyzed and plotted using Seurat (v5.0.3),[44] scRepertoire,[45] or scplotter package (https://github.com/pwwang/scplotter) in RStudio (v2023.06.1; R version 4.3.1 [2023-06-16]). Integration of the single samples were performed using Harmony.[46] To detect CAR T-cell-specific sequences within the data, we created a custom reference genome based on the applied CAR T construct sequences using the cellranger mkref pipeline. Mean cluster expression per feature was calculated using Seurat's *AggregateExpression* function. Gene set scores were calculated and plotted using the UCell package.[47] Heatmaps were generated using the pheatmap package. DEGs were identified using Seurat's *FindMarkers* function (test.use = "wilcox", min.pct = 0.1, logfc.threshold = 0.1) and visualized using the EnhancedVolcano package. GSEAs were performed using the fgsea package (version 1.26.0). Human Hallmark category gene sets (e.g., KEGG and GO) were obtained from the Molecular Signatures Database (MSigDB) using the msigdbr R package. DEGs were ranked based on their average log2 fold change, and the resulting gene list served as input for the GSEA analysis.

RNA sequencing

Activated T cells were mono-transfected with either the SB mRNA or one of the CD19/R110 CAR constructs in duplicates using the same electroporation and expansion process as described for the generation of CAR T cells. After 14 days, total RNA was isolated using the Quick-RNA kit (Zymo Research) and reverse transcribed with SuperScript III (Thermo Fisher Scientific). RNA integrity was tested on a TapeStation (Agilent Technologies), and concentration was quantified using the Qubit platform (QIAGEN). Library preparation and sequencing was performed by Novogene as service. Raw reads were mapped to the human genome (gencode.v44) with SALMON (v.1.10.3)[48] and the read counts processed using package DESeq2 (v.1.48.1)[49] in Rstudio (R version 4.5.0). All CAR mono-transfections were pooled as replicates. Pathway analysis was done with package enrichR, and volcano plot was visualized using package EnhancedVolcano.

DATA AVAILABILITY

Single-cell sequencing data have been deposited at ENA under the accession number PRJEB92038. Any additional data are available from the corresponding authors upon request.

ACKNOWLEDGMENTS

We thank Adeline Stiefvater for outstanding technical support and Mihaela Barbu-Stevanovic, Cécile Cumin, and Morgane Hilpert from the flow cytometry core facility at the Department of Biomedicine at the University Hospital Basel for excellent technical assistance. This study was partially funded by SinABiomedics as well as the innovation focus cell therapies at the University Hospital of Basel, the Swiss National Science Foundation (to S.N.S.F.) (10.001.762) (to M.B.), and the Mertelsmann Foundation (project grant to M.B.).

AUTHOR CONTRIBUTIONS

Idea and design of research project, M.B.; supply of critical material (e.g., patient material, cohorts), M.B. and F.S.; establishment of methods, S.S., C.S., P.B.S., A.Z., C.F., and H.L.; experimental work, C.S., S.S., P.S.B., B.B., M.K., C.F., A.Z., and N.F.; analysis and interpretation of primary data, M.B., C.S., S.S., S.A., and P.B.S.; drafting of manuscript, M.B., C.S., and S.S. All authors reviewed and revised the manuscript.

DECLARATION OF INTERESTS

M.B. is inventor of patent EP22186810.2.

SUPPLEMENTAL INFORMATION

Supplemental information can be found online at https://doi.org/10.1016/j.omtm.2025.101586.

REFERENCES

1. June, C.H., and Sadelain, M. (2018). Chimeric Antigen Receptor Therapy. N. Engl. J. Med. *379*, 64–73. https://doi.org/10.1056/NEJMra1706169.
2. Cappell, K.M., and Kochenderfer, J.N. (2023). Long-term outcomes following CAR T cell therapy: what we know so far. Nat. Rev. Clin. Oncol. *20*, 359–371. https://doi.org/10.1038/s41571-023-00754-1.
3. Gandara, C., Affleck, V., and Stoll, E.A. (2018). Manufacture of Third-Generation Lentivirus for Preclinical Use, with Process Development Considerations for Translation to Good Manufacturing Practice. Hum. Gene Ther. Methods *29*, 1–15. https://doi.org/10.1089/hgtb.2017.098.
4. White, M., Whittaker, R., Gándara, C., and Stoll, E.A. (2017). A Guide to Approaching Regulatory Considerations for Lentiviral-Mediated Gene Therapies. Hum. Gene Ther. Methods *28*, 163–176. https://doi.org/10.1089/hgtb.2017.096.
5. Kebriaei, P., Singh, H., Huls, M.H., Figliola, M.J., Bassett, R., Olivares, S., Jena, B., Dawson, M.J., Kumaresan, P.R., Su, S., et al. (2016). Phase I trials using Sleeping Beauty to generate CD19-specific CAR T cells. J. Clin. Investig. *126*, 3363–3376. https://doi.org/10.1172/JCI86721.
6. Maiti, S.N., Huls, H., Singh, H., Dawson, M., Figliola, M., Olivares, S., Rao, P., Zhao, Y.J., Multani, A., Yang, G., et al. (2013). Sleeping beauty system to redirect T-cell specificity for human applications. J. Immunother. *36*, 112–123. https://doi.org/10.1097/CJI.0b013e3182811ce9.
7. Diez, B., Calviño, C., Fernández-García, M., Rodríguez-Márquez, P., Rodríguez-Díaz, S., Martínez-Turillas, R., Ceballos, C., Illarramendi, J., Serrano-López, J., Miskey, C., et al. (2025). Generation and GMP scale-up of human CAR-T cells using non-viral Sleeping Beauty transposons for B cell malignances. Mol. Ther. Methods Clin. Dev. *33*, 101425. https://doi.org/10.1016/j.omtm.2025.101425.
8. Kouro, T., Himuro, H., and Sasada, T. (2022). Exhaustion of CAR T cells: potential causes and solutions. J. Transl. Med. *20*, 239. https://doi.org/10.1186/s12967-022-03442-3.
9. Fraietta, J.A., Lacey, S.F., Orlando, E.J., Pruteanu-Malinici, I., Gohil, M., Lundh, S., Boesteanu, A.C., Wang, Y., O'Connor, R.S., Hwang, W.T., et al. (2018). Determinants of response and resistance to CD19 chimeric antigen receptor (CAR) T cell therapy of chronic lymphocytic leukemia. Nat. Med. *24*, 563–571. https://doi.org/10.1038/s41591-018-0010-1.
10. Long, A.H., Haso, W.M., Shern, J.F., Wanhainen, K.M., Murgai, M., Ingaramo, M., Smith, J.P., Walker, A.J., Kohler, M.E., Venkateshwara, V.R., et al. (2015). 4-1BB costimulation ameliorates T cell exhaustion induced by tonic signaling of chimeric antigen receptors. Nat. Med. *21*, 581–590. https://doi.org/10.1038/nm.3838.
11. Gomes-Silva, D., Mukherjee, M., Srinivasan, M., Krenciute, G., Dakhova, O., Zheng, Y., Cabral, J.M.S., Rooney, C.M., Orange, J.S., Brenner, M.K., and Mamonkin, M. (2017). Tonic 4-1BB Costimulation in Chimeric Antigen Receptors Impedes T Cell Survival and Is Vector-Dependent. Cell Rep. *21*, 17–26. https://doi.org/10.1016/j.celrep.2017.09.015.
12. Watanabe, N., Bajgain, P., Sukumaran, S., Ansari, S., Heslop, H.E., Rooney, C.M., Brenner, M.K., Leen, A.M., and Vera, J.F. (2016). Fine-tuning the CAR spacer improves T-cell potency. OncoImmunology *5*, e1253656. https://doi.org/10.1080/2162402X.2016.1253656.
13. Ajina, A., and Maher, J. (2018). Strategies to Address Chimeric Antigen Receptor Tonic Signaling. Mol. Cancer Ther. *17*, 1795–1815. https://doi.org/10.1158/1535-7163.MCT-17-1097.
14. Markl, F., Schultheiss, C., Ali, M., Chen, S.S., Zintchenko, M., Egli, L., Mietz, J., Chijioke, O., Paschold, L., Spajic, S., et al. (2024). Mutation-specific CAR T cells as precision therapy for IGLV3-21(R110) expressing high-risk chronic lymphocytic leukemia. Nat. Commun. *15*, 993. https://doi.org/10.1038/s41467-024-45378-w.
15. Lopez-Cantillo, G., Uruena, C., Camacho, B.A., and Ramirez-Segura, C. (2022). CAR-T Cell Performance: How to Improve Their Persistence? Front. Immunol. *13*, 878209. https://doi.org/10.3389/fimmu.2022.878209.
16. Chan, J.D., Lai, J., Slaney, C.Y., Kallies, A., Beavis, P.A., and Darcy, P.K. (2021). Cellular networks controlling T cell persistence in adoptive cell therapy. Nat. Rev. Immunol. *21*, 769–784. https://doi.org/10.1038/s41577-021-00539-6.
17. Magnani, C.F., Gaipa, G., Lussana, F., Belotti, D., Gritti, G., Napolitano, S., Matera, G., Cabiati, B., Buracchi, C., Borleri, G., et al. (2020). Sleeping Beauty-engineered CAR T cells achieve antileukemic activity without severe toxicities. J. Clin. Investig. *130*, 6021–6033. https://doi.org/10.1172/JCI138473.
18. Salmon, A.J., Shavkunov, A.S., Miao, Q., Jarjour, N.N., Keshari, S., Esaulova, E., Williams, C.D., Ward, J.P., Highsmith, A.M., Pineda, J.E., et al. (2022). BHLHE40 Regulates the T-Cell Effector Function Required for Tumor Microenvironment Remodeling and Immune Checkpoint Therapy Efficacy. Cancer Immunol. Res. *10*, 597–611. https://doi.org/10.1158/2326-6066.CIR-21-0129.
19. Qin, Y., Wang, M., Meng, X., Wang, M., Jiang, H., Gao, Y., Li, J., Zhao, C., Han, C., Zhao, W., and Zheng, X. (2024). ISGylation by HERCs facilitates STING activation. Cell Rep. *43*, 114135. https://doi.org/10.1016/j.celrep.2024.114135.
20. Davidson, D., Bakinowski, M., Thomas, M.L., Horejsi, V., and Veillette, A. (2003). Phosphorylation-dependent regulation of T-cell activation by PAG/Cbp, a lipid raft-associated transmembrane adaptor. Mol. Cell Biol. *23*, 2017–2028. https://doi.org/10.1128/MCB.23.6.2017-2028.2003.

21. Saibil, S.D., Deenick, E.K., and Ohashi, P.S. (2007). The sound of silence: modulating anergy in T lymphocytes. Curr. Opin. Immunol. *19*, 658–664. https://doi.org/10.1016/j.coi.2007.08.005.

22. Barili, V., Fisicaro, P., Montanini, B., Acerbi, G., Filippi, A., Forleo, G., Romualdi, C., Ferracin, M., Guerrieri, F., Pedrazzi, G., et al. (2020). Targeting p53 and histone methyltransferases restores exhausted CD8+ T cells in HCV infection. Nat. Commun. *11*, 604. https://doi.org/10.1038/s41467-019-14137-7.

23. Duan, X., Hu, J., Zhang, Y., Zhao, X., Yang, M., Sun, T., Liu, S., Chen, X., Feng, J., Li, W., et al. (2024). RIG-I is an intracellular checkpoint that limits CD8(+) T-cell antitumour immunity. EMBO Mol. Med. *16*, 3005–3025. https://doi.org/10.1038/s44321-024-00136-9.

24. Peters, F.S., Strefford, J.C., Eldering, E., and Kater, A.P. (2021). T-cell dysfunction in chronic lymphocytic leukemia from an epigenetic perspective. Haematologica *106*, 1234–1243. https://doi.org/10.3324/haematol.2020.267914.

25. van Bruggen, J.A.C., Peters, F.S., Mes, M., Rietveld, J.M., Cerretani, E., Cretenet, G., van Kampen, R., Jongejan, A., Moerland, P.D., Melenhorst, J.J., et al. (2024). T-cell dysfunction in CLL is mediated through expression of Siglec-10 ligands CD24 and CD52 on CLL cells. Blood Adv. *8*, 4633–4646. https://doi.org/10.1182/bloodadvances.2023011934.

26. Camerini, E., Amsen, D., Kater, A.P., and Peters, F.S. (2024). The complexities of T-cell dysfunction in chronic lymphocytic leukemia. Semin. Hematol. *61*, 163–171. https://doi.org/10.1053/j.seminhematol.2024.04.001.

27. Vlachonikola, E., Stamatopoulos, K., and Chatzidimitriou, A. (2020). T Cells in Chronic Lymphocytic Leukemia: A Two-Edged Sword. Front. Immunol. *11*, 612244. https://doi.org/10.3389/fimmu.2020.612244.

28. Roessner, P.M., and Seiffert, M. (2020). T-cells in chronic lymphocytic leukemia: Guardians or drivers of disease? Leukemia *34*, 2012–2024. https://doi.org/10.1038/s41375-020-0873-2.

29. Niu, A., Zou, J., Hu, X., Zhang, Z., Su, L., Wang, J., Lu, X., Zhang, W., Chen, W., and Zhang, X. (2023). Differences in the phenotypes and transcriptomic signatures of chimeric antigen receptor T lymphocytes manufactured via electroporation or lentiviral transfection. Front. Immunol. *14*, 1068625. https://doi.org/10.3389/fimmu.2023.1068625.

30. Turtle, C.J., Hanafi, L.A., Berger, C., Gooley, T.A., Cherian, S., Hudecek, M., Sommermeyer, D., Melville, K., Pender, B., Budiarto, T.M., et al. (2016). CD19 CAR-T cells of defined CD4+:CD8+ composition in adult B cell ALL patients. J. Clin. Investig. *126*, 2123–2138. https://doi.org/10.1172/JCI85309.

31. Sommermeyer, D., Hudecek, M., Kosasih, P.L., Gogishvili, T., Maloney, D.G., Turtle, C.J., and Riddell, S.R. (2016). Chimeric antigen receptor-modified T cells derived from defined CD8+ and CD4+ subsets confer superior antitumor reactivity in vivo. Leukemia *30*, 492–500. https://doi.org/10.1038/leu.2015.247.

32. Xu, Y., Zhang, M., Ramos, C.A., Durett, A., Liu, E., Dakhova, O., Liu, H., Creighton, C.J., Gee, A.P., Heslop, H.E., et al. (2014). Closely related T-memory stem cells correlate with in vivo expansion of CAR.CD19-T cells and are preserved by IL-7 and IL-15. Blood *123*, 3750–3759. https://doi.org/10.1182/blood-2014-01-552174.

33. Watson, H.A., Durairaj, R.R.P., Ohme, J., Alatsatianos, M., Almutairi, H., Mohammed, R.N., Vigar, M., Reed, S.G., Paisey, S.J., Marshall, C., et al. (2019). L-Selectin Enhanced T Cells Improve the Efficacy of Cancer Immunotherapy. Front. Immunol. *10*, 1321. https://doi.org/10.3389/fimmu.2019.01321.

34. Kasuya, H., Zhang, H., Ito, Y., Yoshikawa, T., Nakashima, T., Li, Y., Matsukawa, T., Inoue, S., and Kagoya, Y. (2024). High CD62L expression predicts the generation of chimeric antigen receptor T cells with potent effector functions. Int. Immunol. *36*, 353–364. https://doi.org/10.1093/intimm/dxae015.

35. Brown, C.E., Hibbard, J.C., Alizadeh, D., Blanchard, M.S., Natri, H.M., Wang, D., Ostberg, J.R., Aguilar, B., Wagner, J.R., Paul, J.A., et al. (2024). Locoregional delivery of IL-13Ralpha2-targeting CAR-T cells in recurrent high-grade glioma: a phase 1 trial. Nat. Med. *30*, 1001–1012. https://doi.org/10.1038/s41591-024-02875-1.

36. DeGolier, K.R., Danis, E., D'Antonio, M., Cimons, J., Yarnell, M., Kedl, R.M., Kohler, M.E., Scott-Browne, J.P., and Fry, T.J. (2025). Antigen experience history directs distinct functional states of CD8(+) CAR T cells during the antileukemia response. Nat. Immunol. *26*, 68–81. https://doi.org/10.1038/s41590-024-02034-1.

37. Wherry, E.J., and Kurachi, M. (2015). Molecular and cellular insights into T cell exhaustion. Nat. Rev. Immunol. *15*, 486–499. https://doi.org/10.1038/nri3862.

38. Webster, B., Xiong, Y., Hu, P., Wu, D., Alabanza, L., Orentas, R.J., Dropulic, B., and Schneider, D. (2021). Self-driving armored CAR-T cells overcome a suppressive milieu and eradicate CD19(+) Raji lymphoma in preclinical models. Mol. Ther. *29*, 2691–2706. https://doi.org/10.1016/j.ymthe.2021.05.006.

39. Hudecek, M., Sommermeyer, D., Kosasih, P.L., Silva-Benedict, A., Liu, L., Rader, C., Jensen, M.C., and Riddell, S.R. (2015). The nonsignaling extracellular spacer domain of chimeric antigen receptors is decisive for in vivo antitumor activity. Cancer Immunol. Res. *3*, 125–135. https://doi.org/10.1158/2326-6066.CIR-14-0127.

40. Weber, K., Bartsch, U., Stocking, C., and Fehse, B. (2008). A multicolor panel of novel lentiviral "gene ontology" (LeGO) vectors for functional gene analysis. Mol. Ther. *16*, 698–706. https://doi.org/10.1038/mt.2008.6.

41. Belkina, A.C., Ciccolella, C.O., Anno, R., Halpert, R., Spidlen, J., and Snyder-Cappione, J.E. (2019). Automated optimized parameters for T-distributed stochastic neighbor embedding improve visualization and analysis of large datasets. Nat. Commun. *10*, 5415. https://doi.org/10.1038/s41467-019-13055-y.

42. Schultheiß, C., Paschold, L., Willscher, E., Simnica, D., Wöstemeier, A., Muscate, F., Wass, M., Eisenmann, S., Dutzmann, J., Keyßer, G., et al. (2021). Maturation trajectories and transcriptional landscape of plasmablasts and autoreactive B cells in COVID-19. iScience *24*, 103325. https://doi.org/10.1016/j.isci.2021.103325.

43. Schultheiß, C., Paschold, L., Mohebiany, A.N., Escher, M., Kattimani, Y.M., Müller, M., Schmidt-Barbo, P., Mensa-Vilaró, A., Aróstegui, J.I., Boursier, G., et al. (2024). A20 haploinsufficiency disturbs immune homeostasis and drives the transformation of lymphocytes with permissive antigen receptors. Sci. Adv. *10*, eadl3975. https://doi.org/10.1126/sciadv.adl3975.

44. Hao, Y., Hao, S., Andersen-Nissen, E., Mauck, W.M., 3rd, Zheng, S., Butler, A., Lee, M.J., Wilk, A.J., Darby, C., Zager, M., et al. (2021). Integrated analysis of multimodal single-cell data. Cell *184*, 3573–3587.e29. https://doi.org/10.1016/j.cell.2021.04.048.

45. Borcherding, N., Bormann, N.L., and Kraus, G. (2020). scRepertoire: An R-based toolkit for single-cell immune receptor analysis. F1000Res. *9*, 47. https://doi.org/10.12688/f1000research.22139.2.

46. Korsunsky, I., Millard, N., Fan, J., Slowikowski, K., Zhang, F., Wei, K., Baglaenko, Y., Brenner, M., Loh, P.R., and Raychaudhuri, S. (2019). Fast, sensitive and accurate integration of single-cell data with Harmony. Nat. Methods *16*, 1289–1296. https://doi.org/10.1038/s41592-019-0619-0.

47. Andreatta, M., and Carmona, S.J. (2021). UCell: Robust and scalable single-cell gene signature scoring. Comput. Struct. Biotechnol. J. *19*, 3796–3798. https://doi.org/10.1016/j.csbj.2021.06.043.

48. Patro, R., Duggal, G., Love, M.I., Irizarry, R.A., and Kingsford, C. (2017). Salmon provides fast and bias-aware quantification of transcript expression. Nat. Methods *14*, 417–419. https://doi.org/10.1038/nmeth.4197.

49. Love, M.I., Huber, W., and Anders, S. (2014). Moderated estimation of fold change and dispersion for RNA-seq data with DESeq2. Genome Biol. *15*, 550. https://doi.org/10.1186/s13059-014-0550-8.

OMTM, Volume *33*

Supplemental information

CAR T cell engineering impacts antigen-independent activation and co-inhibition

Christoph Schultheiß, Simon Stücheli, Brenda Besemer, Maja Kadel, Paul Schmidt-Barbo, Andreas Zingg, Natascha Franz, Sarah Adamo, Frank Stenner, Claudia Fischer, Heinz Läubli, and Mascha Binder

Table S1. Antibodies used for flow cytometry.

Target	Conjugated fluorophore	Supplier	Reference Number	Host species	Comment
EGFRt	NA	Merck (Darmstadt, Germany)	NA (Erbitux®)	NA (Humanized)	Cetuximab, used to detect the spliced off truncated EGFR as a surrogate for the CAR construct. Diluted at 1:500.
Human IgG	FITC	Thermo Fisher Scientific	F4512-1ML	Rabbit	Used to detect Cetuximab/anti-EGFR-antibody. Diluted at 1:1000.
Whitlow/218-scFv-Linker	NA	Cell Signaling Technologies (Danvers, MA, USA)	57710S	Rabbit	Used to detect anti-CD19 CAR.
G4S-scFv-Linker	NA	Cell Signaling Technologies	71645S	Rabbit	Used to detect anti-R110 CAR.
Rabbit IgG	AF488	Cell Signaling Technologies	4412S	Goat	Used to detect anti-scFv-Linker-antibodies.
CD19	PE/Dazzle	BioLegend (San Diego, CA, USA)	302251	Mouse	
CD19	APC	BioLegend	302212	Mouse	
CD3	AF700	BioLegend	300424	Mouse	
CD3	FITC	BD BioSciences (Franklin Lanes, NJ, USA)	345763	Mouse	Used for sorting prior to single-cell sequencing.
CD4	BV605	BioLegend	344646	Mouse	
CD8	BV650	BioLegend	344729	Mouse	
PD-1	PE	BioLegend	329905	Mouse	
LAG-3	PE/Cy7	BioLegend	369309	Mouse	
TIM-3	APC/Cy7	BioLegend	345025	Mouse	
CD69	APC	BioLegend	985206	Mouse	
CCR7	PerCP	Invitrogen (Carlsbad, CA, USA)	46-1979-42	Rat	
CD45RA	APC	Invitrogen	47-0458-42	Mouse	
IGLV3-21^{R110} light chain	PE	AVA LifeScience (Denzlingen, Germany)	AVA-D01-PE/01P0150	Mouse	

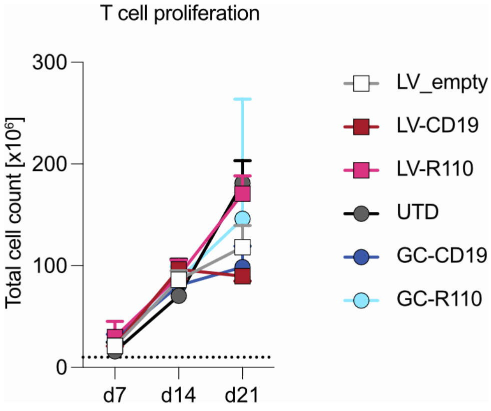

Figure S1. Proliferation of T cells in CAR expansion cultures over time. Proliferation quantified in HD-derived batches (n=3) and shown as mean ± SD. Dotted line indicates seeding amount.

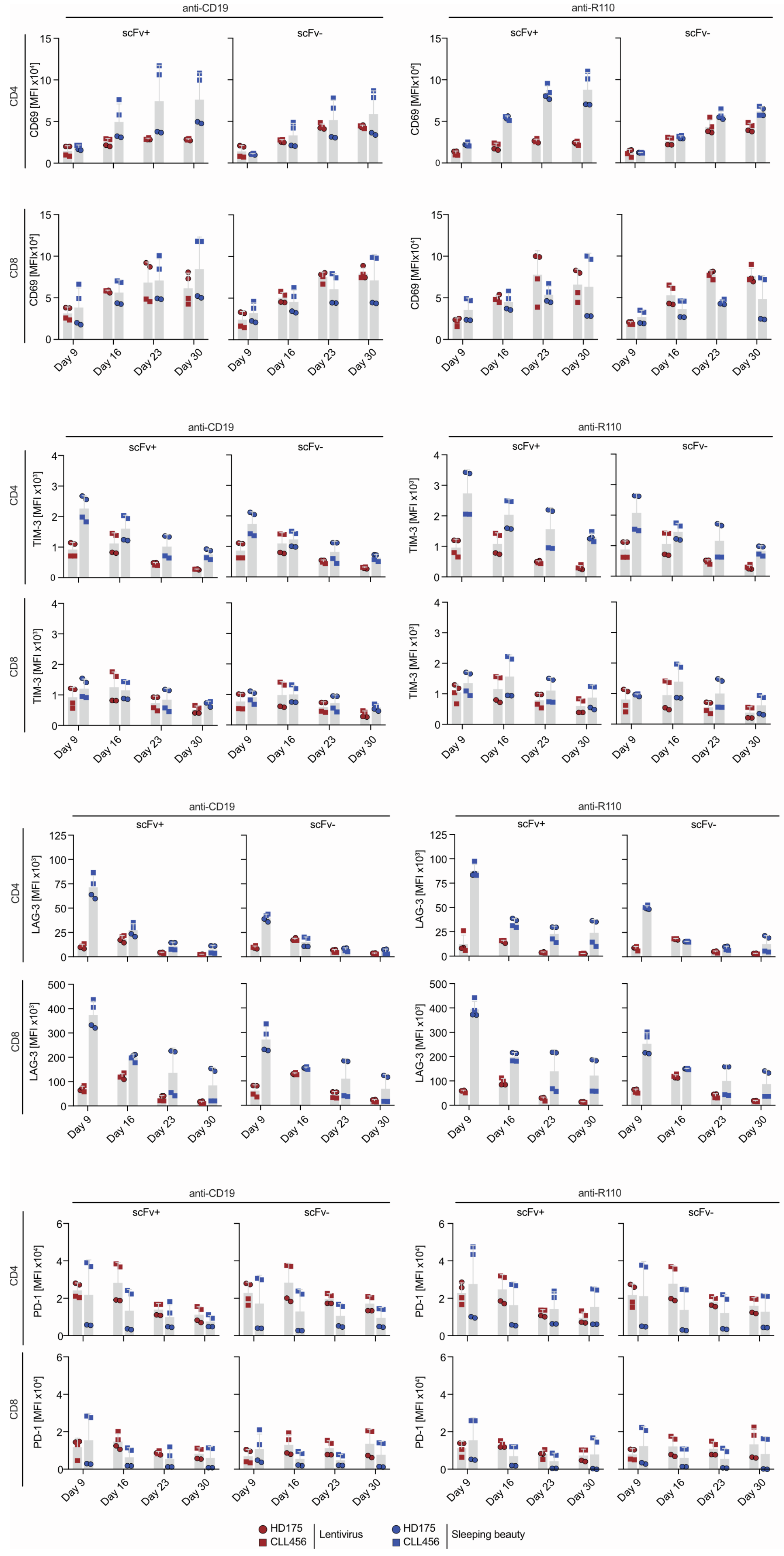
anti-CD19
anti-R110
scFv+
scFv-
CD4
CD8
CD69 [MFI x10⁴]
TIM-3 [MFI x10³]
LAG-3 [MFI x10³]
PD-1 [MFI x10⁴]
Day 9
Day 16
Day 23
Day 30
HD175
CLL456
Lentivirus
HD175
CLL456
Sleeping beauty

Figure S2. Expression patterns of activation and co-inhibition markers in healthy donor and CLL patient-derived CAR T cells by multicolor flow cytometric analysis. (A) CD69, **(B)** TIM-3, **(C)** LAG-3 and **(D)** PD-1 mean fluorescence intensities (MFI) in CAR T cells separated by CD4, CD8, scFv$^+$ and scFv$^-$ expression. Bars represent the mean ± SD, each data point represents an individual donor.

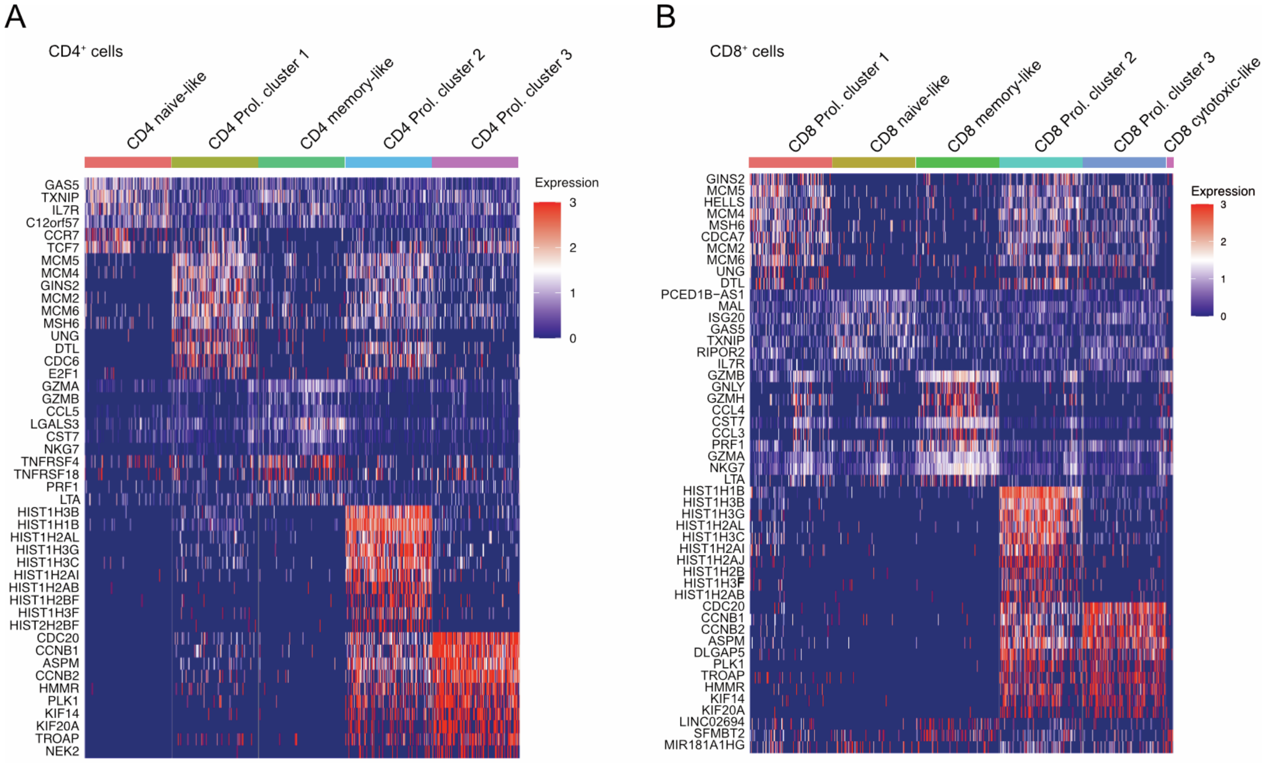

Figure S3. Top 10 differentially expressed genes per cluster in the CD4⁺ (A) and CD8⁺ (B) subsets.

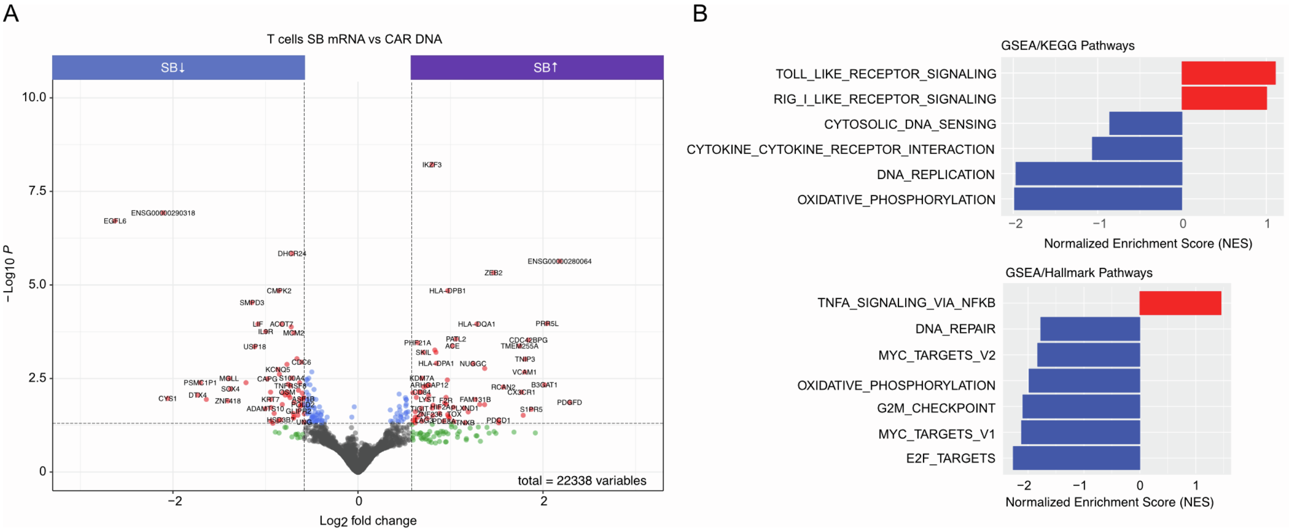

Figure S4. Characterization of transcriptomic signatures in T cells transfected with SB mRNA or CAR DNA. (A) Volcano plot showing genes differentially expressed genes (DEGs; adjusted P < 0.01) between T cells at day 14 post-transfection with either SB mRNA or CAR DNA alone. **(B)** Gene set enrichment of GSEA/KEGG (upper panel) and GSEA/Hallmark pathways (lower panel) for DEGs from (A).

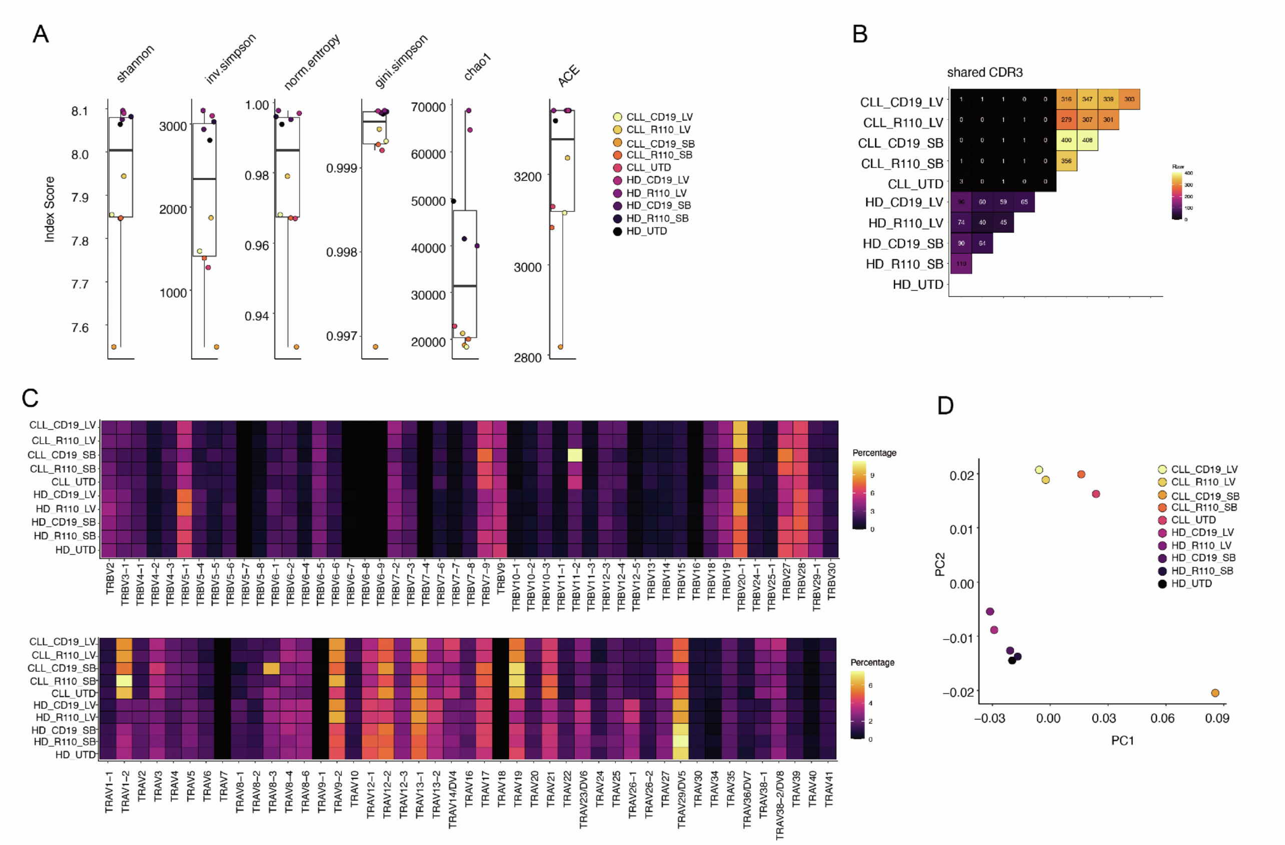

Figure S5. T cell receptor repertoire analysis of SB- and LV-manufactured CAR T cells. (A) Immune metrics. **(B)** Shared CDR3 sequences between donors and production batches. **(C)** Frequencies of TRBV gene usage. **(D)** PCA of TRBVJ usage.
